# Supplementary material for: Noninvasive electromagnetic source imaging of spatiotemporally distributed epileptogenic brain sources
Source: Nat Commun. 2020 Apr 23;11:1946. doi: 10.1038/s41467-020-15781-0 (PMC7181775; doi:10.1038/s41467-020-15781-0)
Supplement: Supplementary file 1 — Supplementary Information [file 41467_2020_15781_MOESM1_ESM.pdf]

Supplementary Information File

For

Noninvasive Electromagnetic Source Imaging of Spatio-temporally Distributed Epileptogenic Brain Sources

Sohrabpour et al.

Supplementary Note 1: A Spatio-temporal Approach Towards Source Imaging

The iteratively reweighted edge sparsity minimization strategy (IRES), can be used to image the brain electrical activity over time and provide a dynamic image of underlying brain activity. One application of IRES is to perform dynamic source imaging as opposed to solving the inverse electromagnetic source imaging (ESI) problem for every time point. The scalp potential (or magnetic field) measurements over the interval to be studied can be decomposed into spatial and temporal components using the independent component analysis (ICA) and/or any other blind source separation (BSS) technique. In this manner, a temporal basis can be formed for the underlying sources. Basically, we would like to model the underlying electrical activity of the source as spatial components that have coherent activity over time. In this manner, we are decomposing the brain into multiple regions where each region has its specific and coherent activity over time, mathematically speaking:

$$\mathbf{j}(t) = \sum_{i=1}^{N_c} \mathbf{j}_i \otimes \mathbf{a}_i(t) \tag{1}$$

Where $\mathbf{j}(t)$ is the underlying electrical activity of the brain over time (an N×T matrix where N is the number of current sources in the brain and T is the number of time points within the time interval of interest in the potential/field recordings), $\mathbf{j}_i$ is the activity of region i, within the brain (an N×1 vector), with its corresponding activation over time, $\mathbf{a}_i(t)$ (an 1×T vector). The symbol ⊗ represents an outer product operator. N_c is the number of active regions. If the time-course of underlying sources' activity can be determined from the scalp measurements, then the IRES optimization problem can be solved to determine the $\mathbf{j}_i$'s ($\mathbf{j} = [j_1, j_2, \dots]$), as follows:

$$\mathbf{j}^L = \underset{\mathbf{j}}{argmin} \sum_{i=1}^{N_c} \parallel \mathbf{W}_{d,i}^{L-1}(\mathbf{V}\mathbf{j}_i) \parallel_1 + \alpha \sum_{i=1}^{N_c} \parallel \mathbf{W}_i^{L-1}\mathbf{j}_i \parallel_1$$

$$subject\ to \quad \mathrm{Trace}\{(\boldsymbol{\phi}(t) - \mathbf{KjA})^T \boldsymbol{\Sigma}^{-1} (\boldsymbol{\phi}(t) - \mathbf{KjA})\} \leq \beta^2 \tag{2}$$

Where $\boldsymbol{\phi}(t)$ is the scalp potential (or magnetic field) measurement over the interval of interest (an E×T matrix where E is the number of measurements), $\mathbf{K}$ is the lead field matrix (an E×N matrix), $\mathbf{j}$ is the unknown current density of the brain regions (an N× N_c matrix), $\mathbf{A}$ is the time course activation matrix (an N_c ×T matrix) which is given by, $\mathbf{A} = [\mathbf{a}_1(t), \mathbf{a}_2(t), \ldots]$, β^2 is essentially the noise power, to be determined by the discrepancy theorem, $\boldsymbol{\Sigma}$ is the covariance matrix of the noise to be determined from the baseline activity, $\mathbf{W}_{d,i}^{L-1}$ and $\mathbf{W}_i^{L-1}$ are the weights pertaining to each $\mathbf{j}_i$ and are updated with the same rule determined for the IRES, $\mathbf{V}$ is the discrete gradient operator, α is the hyper-parameter balancing between the two terms in the regularization term which will be tuned using the L-curve approach and L is counting the iteration steps. The component analysis will be used to estimate the $\mathbf{a}_i(t)$'s (and consequently $\mathbf{A}$) as there is a linear relation between underlying current density distribution $\mathbf{j}$ and the scalp potential/field measurements $\boldsymbol{\phi}$:

$$\boldsymbol{\phi}(t) = \mathbf{Kj}(t) = \mathbf{K} \sum_{i=1}^{N_c} \mathbf{j}_i \otimes \mathbf{a}_i(t) = \sum_{i=1}^{N_c} (\mathbf{Kj}_i) \otimes \mathbf{a}_i(t) = \sum_{i=1}^{N_c} \boldsymbol{\psi}_i \otimes \mathbf{a}_i(t) \quad (3)$$

Where $\boldsymbol{\psi}_i = \mathbf{Kj}_i$. Thus, if the scalp measurements can be decomposed into spatio-temporal components $\boldsymbol{\phi}(t) = \sum_{i=1}^{N_c} \boldsymbol{\phi}_i \otimes \mathbf{a}_i{}^*(t)$, we can extract the time-course activity of underlying sources, in the brain, although the decomposed temporal components $\mathbf{a}_i{}^*(t)$ might not be equal to $\mathbf{a}_i(t)$ in a one-to-one manner, as long as $\mathbf{A}^* = [\mathbf{a}_1{}^*(t), \mathbf{a}_2{}^*(t), \ldots]$ contains the same essential components of $\mathbf{A}$, the analysis will hold,

$$\mathbf{A} = \mathcal{L}\{\mathbf{A}^*\} = \mathbf{LA}^* \quad (4)$$

Where $\mathcal{L}$ is a linear transformation between the two matrices ($\mathbf{L}$ is an $N_c \times N_c$ square matrix). The number of components cannot be determined a priori, but there are two methods to accomplish this goal. One, is to discard components which are noisy and extremely weak not surpassing the noise level (which can be computed from baseline activity in the measurements, for instance). The second approach would be to select components that are either time-locked to the stimuli present in our experiment (external stimuli

such as a flash of light in case of visual evoked responses or internal stimuli such as inter-ictal spike activity or the onset of seizure activity) or to select components which present desirable features in the spectrum such as event related synchronization (ERS) and event related desynchronization (ERD) [1]. In any case, PCA and ICA and BSSA techniques in general, are used extensively in separating desirable and noisy signals, in the signal processing applications and thus provide a powerful framework for us to estimate **A** or the temporal basis [2,3]. It is good to note that the proposed formulation still estimates $\mathbf{j}_i$'s, inverting the effects of volume conduction, as the spatial component is affected by the volume conduction, while the temporal activity, which is not affected by the volume conduction, is estimated from the scalp measurements. The proposed method could also be applied iteratively, to increase accuracy; that is, after the locations and time-course activity of the underlying sources are estimated, the time course activity of each $\mathbf{j}_i$ can be estimated by projecting the scalp measurements to the columns of **K** pertaining to source locations in the estimated $\mathbf{j}_i$ to estimate the corresponding $\mathbf{a}_i(t)$, and then once the temporal basis is formed again, the process of estimating $\mathbf{j}_i$'s can be repeated. In this manner, the spatio-temporal source imaging IRES will improve at every iteration.

Supplementary Note 2: Simulation Study - FAST-IRES

In order to provide a thorough evaluation of FAST-IRES a Monte Carlo simulation was performed. Random locations were selected on the cortex as seed points, around which an extended source was created (115 location on the cortex). The extent size of these sources ranged from 0 mm[†] to around 40 mm (each location was randomly assigned an extent). Once the extended sources were placed on a fine cortical model (66,490 triangular surface elements) the forward problem was solved to generate the scalp potentials of these sources and different levels of noise was added to this simulated EEG to obtain SNR levels of 0, 5, 10, and 20 dB. The noise was a combination of realistic correlated EEG noise (from non-epileptic periods

---

[†] It must be noted that the smallest source possible to place on the cortex is a dipole; a dipole by nature, is a representation of the activity of a small cortical area. In our model this means a single surface tringle, where the dipole is placed at the center is activated (surface triangles tessellating the brain). The area of such triangular elements is about 1.5-3 mm^2 (equivalent to an average radius of 0.7-1 mm).

of EEG recordings) and additive white Gaussian noise (equal power to realistic EEG noise). For each simulation case for a given an SNR, 10 noise realizations were analyzed (accruing to a total of 1,150 cases for each SNR level). The inverse was then subsequently calculated for these noisy scalp maps on a coarse cortical grid (31,530 triangular surface elements). The extent of the estimated sources was compared against the simulated sources extent and plotted against each other (Fig. 2 in the main body of this paper). Calculating the Pearson's correlational value and its related p-value, we found statistically significant and high correlational values, proving that our method robustly estimates underlying sources' extents (refer to Supplementary Table 1 for quantitative and more detailed results).

It must be mentioned that as is evident in the results presented in Fig. 2 of the main text, our estimates show the stochastic nature of our algorithm (as in almost any source imaging algorithm), as our estimates do not fall exactly on a straight line. This variation is partly due to noise and partly due to the geometrical complexities of the cortex, specifically for larger sources. Supplementary Fig. 1 shows some simulation examples.

Supplementary Fig. 2 depicts the application of FAST-IRES in a computer simulation example. As the example indicates, FAST-IRES is successful in efficiently and accurately determining the underlying brain networks. Imaging epilepsy networks is only one application of this approach and potentially many spatio-temporal underlying brain activities can be monitored using the proposed approach. In order to test the capability of this formulation we further tested this method in two simulation examples.

In these examples three sources were simulated. Three sources were randomly chosen in the first example and based on this example a more challenging case, where two of the sources were moved closer (Refer to Supplementary Fig. 3). Each extended source (source sizes are reported in Supplementary Table 3) was assigned a time-course of activity. These time courses contain about 800 samples (128 EEG channels) and are almost uncorrelated (given all the internal iterations and the iterative reweighting iterations proposed in FAST-IRES it takes about 1~2 minutes to obtain solutions). The forward problem

was solved, and different amount of white Gaussian noise were added to the simulated scalp maps from these two examples, resulting in a total signal to noise ratio (SNR) of 5, 10 and 20 dB. The forward and inverse models used were different and similar to the one reported in [4] for the bulk of our simulations; basically, insuring different grid sizes for forward and inverse (much finer for forward and coarser for inverse) computation.

The results are graphically depicted in Supplementary Fig. 3 and the quantitative assessment of the obtained results is summarized in Tables S2 and S3. It can be clearly observed that the estimated sources match the simulated sources in location and estimated size quite well; this conclusion is drawn based on the small localization error and accurate source extent size reported in the two aforementioned tables. Additionally, the estimated time-courses of these sources are highly correlated with the simulated time-courses, indicating the high precision of FAST-IRES in estimating temporal activity of underlying sources.

Two points have to be emphasized regarding the simulations. Firstly, a relatively deep source was simulated on the mesial side of the temporal lobe (Source 1). Estimating deep sources from EEG (and to a greater extent in MEG) is always a challenge and yet this source was recovered, applying sparse signal processing techniques implemented in FAST-IRES. Secondly, in the second example the two sources are within a 20 mm distance from each other (center-to-center) and yet they are distinguished beautifully with precise location and extent size. Even in highly noisy cases when SNR is equal to 5 dB, FAST-IRES can identify deep and proximally located sources quite well. These desirable qualities are important when analyzing real data recorded in EEG and MEG, as indicated in our main results, reported in the main body of this paper.

We proposed a highly efficient algorithm to implement a spatio-temporal approach towards source imaging named FAST-IRES. This algorithm is easy to implement, fast, efficient, and achieves high precisions in terms of localization, estimating extent size of underlying sources and accurately determining

the temporal activity of sources (which is extremely important for a precise estimation of underlying source dynamics and connectivity).

Supplementary Note 3: Connectivity Imaging

One of the main motivations behind our work is to obtain extended solutions that are not dependent on the operator's choice of a threshold. As observed in Fig. 5a (as well as Supplementary Fig. 4), there are regions in the vicinity or further away from the SOZ, that are simultaneously activated. Our approach for ictal imaging is to filter the results at each seizure's dominant frequency and select the region that shows the highest amplitude, as the estimated EZ. This however, might raise an objection; is it fair to disregard regions that have smaller amplitudes? how reliable is this approach? While our findings as well as previous literature supports the approach that we have adopted in localizing the EZ, finding a better approach to more objectively determine the generator region among all other activated regions is desirable.

Solutions obtained from FAST-IRES are piecewise continuous with clear distinction (edges) between signal and background activity without the application of any threshold. Directed connectivity analysis can be applied to the extracted time-course of these regions to determine causal relationships between the activity of certain nodes or brain regions. The idea of applying directed connectivity to source imaging results to determine the nodes which drive the seizure has been investigated previously [5–7] and has proven to be of merit. In this part of the study a similar line of investigation is adopted, with the distinction that FAST-IRES objectively produces spatially extended distributions that will be designated as nodes in our network analysis, while conventional methods either used dipole solutions or applied arbitrary thresholds to form their network nodes. Effectively, nodes in the network are not necessarily focal points, as one might conceive upon hearing the term node, but spatially extended regions, as one would expect from generators of EEG in the human brain.

The process of extracting nodes is a bit complicated, as for each TBF or time-course of activity, we might find that multiple regions are activated. This means that a particular region might involve multiple

TBFs (our estimated solution where each column corresponds to one row of TBF). In these cases, each region must be separated and treated as a different node. This process, however, results in a large number of regions with highly corelated temporal activity. Thus, a spatio-temporal clustering process was developed to combine the regions which are correlated into hyper-nodes (collection of multiple nodes). Supplementary Fig. 5 provides a schematic diagram that helps better visualize the process. The time-course of these hyper-nodes are finally extracted and the directed transfer function (DTF) analysis was performed on these time-courses to find the causal relationships between nodes and hypernodes. Nodes causally driving the activity of other network nodes were termed driving nodes and were designated as the estimated EZ. This process is schematically pictured in Supplementary Fig. 6a. Note the lack of spatial variation within the distinct regions estimated by FAST-IRES compared to those presented in Fig. 4 and Fig. 5, where the signal's energy in source space was presented and as a result, variations of amplitude along the source extent are evident.

Examples of estimated EZ and the corresponding clinical findings in some patients are presented in Supplementary Fig. 6b. Analytical results such as precision, recall and localization error are presented in Supplementary Fig. 6c. High precision and recall values (about 80%) and localization errors of about 5mm indicate how reliably the EZ can be estimated using the proposed approach. While results obtained from this connectivity imaging method are not statistically superior to ictal imaging results, they are obtained without thresholds. Ital imaging and connectivity imaging results bore no statistically significant results, as indicated by Supplementary Fig. 7 and Supplementary Table 6.

Hyper-node Analysis

Partitioning the source space based on the FAST-IRES solution is a key concept in the process of forming the estimated EZ. First it is necessary to note that FAST-IRES estimates a spatial distribution (in source space) for each row of the TBF (pertaining to columns of FAST-IRES solution, i.e. columns of **j**). Second, the FAST-IRES solution is piecewise homogenous with clear edges. Based on these facts, what needs to be done is to treat the non-zero amplitudes within the spatial distribution of each column of the

IRES solution, as 1 and the rest as 0. Subsequently, for overlapping regions, the overlap was treated as a separate region. This is due to the fact that, if a region is non-zero in more than one column of **j** then more than one-time course of activity is assigned to that region as each column of **j** pertains to one row of the TBF. After this step was completed the source space is partitioned into non-overlapping regions. The mean time-course within each region was then extracted. The number of regions, after this process, could be more than the number of TBF rows or independent time-courses; so, in order for the Granger causality analysis to work properly, some of these regions needed to be re-integrated together reducing the number of segments. Simply put, a high correlation was observed among the time-course of activities among these regions. To this end the correlation matrix among the mean time-course of these spatially non-overlapping regions were calculated. Regions that were spatially adjacent (neighboring regions) and demonstrated high correlation during the initial seizure phase, were integrated together. Non-neighboring regions with high correlations (a rare event) were not integrated together and treated as separate regions (unless the correlation was perfect, that is 1 or -1). The process of combining individual nodes into hyper-nodes, as described here, is fully automatic, using hierarchical clustering, where the number of clusters can be set, a priori, to the number of TBFs. We used MATLAB's *cluster* function, to this end.

This procedure is depicted in Supplementary Fig. 5 for one of the analyzed patients. An interesting observation is that if we inspect the correlation matrix (the absolute value of the correlation matrix) of the time-course of activities in the original segmentation, we observe that many entries are highly correlated with each other (hotter colors). However, once the correlation matrix is re-indexed and clustered such that these highly correlated nodes are placed next to each other in the matrix, hyper-nodes are formed; nodes that have high correlations among themselves and much less cross-correlations with other nodes. In other words, we can form almost a block diagonal matrix. Basically, we decided to integrate all the nodes within a hyper-node, as a single node; with the exception that if the nodes were not spatially adjacent and the correlations were not perfect, i.e. 1 or -1, they were treated as independent nodes. Please refer to Supplementary Fig. 5 and Supplementary Fig. 6a for more details and better visualization.

At the end of this process a spatio-temporal segmentation was achieved, where adjacent regions with highly similar temporal evolution were placed in a single segment. In this process no threshold was applied to our solutions. This spatio-temporal analysis of spatially extended sources to determine regions of interest (nodes) that would further be analyzed with directed connectivity measures, is another unique and notable feature of our approach, unprecedented in the literature, to the best of our knowledge. This objective, spatio-temporal clustering and forming hyper-nodes would have been almost impossible (without applying thresholds to sources), were it not for the fact that FAST-IRES solutions do not apply subjective thresholds to separate activity from background.

After the new spatially non-overlapping and temporally-coherent regions were formed, the mean time-course of activity was extracted. These time-courses were fed into the DTF connectivity analysis to determine the driving nodes, i.e. those nodes which demonstrated high information outflow.

Implementation

After the spatio-temporal distribution is obtained using FAST-IRES, the source is partitioned into spatially distinct segments. DTF analysis was used to derive directional connectivity in this study [8]. The time-course of activity for each of these regions is extracted and the DTF coefficients are calculated for these regions of interest, typically, in a 0.5 – 1 second interval around the seizure onset time (typically, from around 0.5 second prior to seizure onset to 0.5 second after seizure onset). The DTF was calculated in the 1-15 Hz frequency band as ictal signals recorded in EEG are generally low-frequency [6]. The model order (for the multi-variate auto-regressive model) was selected based on the Akaike information criterion (AIC) [9]. The average DTF for the entire frequency bands was calculated at the seizure onset. The region(s) which has the maximum outflow (sum of columns in the average DTF matrix) was designated as the estimated EZ. Once this region is identified, it is compared to clinical findings. The overall procedure for patient data analysis is schematically depicted in Supplementary Fig. 6a.

Detailed information about the mathematical definition of DTF and how it should be used for analyzing seizure data can be found in our previous work and references therein [7]; however, a few points must be mentioned here. As the relation between the DTF and the time series are highly nonlinear, a nonparametric statistical testing was employed to reject spurious connections due to random noise [10,11]. The surrogate data were generated by keeping the amplitude of the time series' spectrum the same as the original data but permuting the phases. This shuffling procedure was performed 1000 times and if the DTF value computed passed the significance level ($p<0.05$), they were kept, otherwise were replaced by zero [5,6,12]. This shuffling process has been performed for all DTF analysis presented in this paper. The DTF analysis was performed using eConnectome [13] which is an open source software freely available at (https://www.nitrc.org/projects/econnectome/).

A Cautionary Note on Potential Limitations

While source imaging reduces the effect of volume conduction and is shown to delineate underlying brain sources [6,7,14], it is not easy to ascertain that this goal has been fully achieved. FAST-IRES simulation results indicate that this is a strong possibility. Our initial experiment, correlating superficial and deep iEEG recordings with estimated time-courses from FAST-IRES (these findings must be considered with caution as we have explained in the main body and in Supplementary Note 7) indicates that this might be a reasonable assumption for FAST-IRES. However, more work has to be done to reach a clear verdict on this issue. Thus, it is possible that connectivity analyses, DTF analysis in this work, might have been affected by residual effects of volume conduction that might not have been thoroughly eliminated by our proposed inverse approach.

It is also important to note that these connectivity analyses are not to be confused with resting-state connectivity analyses performed in fMRI studies or electrophysiological studies. The term connectivity imaging, in this work, refers to directional connectivity of epilepsy networks using DTF analysis.

Supplementary Note 4: Analyzing Consistent Spikes

To further investigate the difference observed between spike and seizure analysis, we compared "consistent" spikes to seizures in our data base. Consistent spikes were defined as spikes that were ipsilateral to the resection side. Our results indicated that no significant differences could be perceived between consistent spikes and seizures at any level, i.e. total population level, seizure-free cohort or non-seizure-free patients. Results and statistics are presented in Supplementary Fig. 9 and Supplementary Table 8. Note please, that this post-hoc analysis was only possible after the availability of clinical results, implying that in practice defining and rejecting inconsistent spikes is a very difficult task (maybe impossible), if further information, such as ictal recordings, are not available.

Supplementary Note 5: Sub-averaging Approach to Spike Imaging

In order to ensure that spike imaging analyses were not biased we followed Aydin et al. [15] in employing a sub-averaging procedure. In conventional dipole fitting studies [16,17], it is observed that due to spike variability, a distribution of fitted dipoles can be achieved, which provides an estimate of the irritative zone, based on the premise that the different observed spikes are arising from the irritative zone, hence the localized dipoles can reveal this underlying zone in the brain.

The intuition behind this approach is to maximize signal SNR by averaging spikes, yet, preserving spike variability by not averaging all the spikes at once and only averaging together randomly drawn sets of spikes (from the total spike population). This simultaneously improves SNR and preserves spike-to-spike variability. In earlier source imaging studies where dipole localization was employed, variability of spikes, resulted in dipoles being localized to different brain regions, hence, resulting in a distribution of dipoles that could reveal the extent of the irritative zone. Our proposed approach is different as it is robust to noise and provides extended solutions which can count for the variability of spikes; moreover, if spikes were different, they would have been categorized as a different spike type and would have been analyzed separately (in our current pipeline). Our analysis also showed that average spike results slightly exceed the mean and median of these random sub-averaged populations (we compared the geometric mean of averaged

spike to the random sub-averaged population). It is important to emphasize that sub-averaging techniques are important to consider when performing spike analyses, specifically, if spike numbers are limited, but in our proposed framework, no significant improvement was observed.

As the SNR of single spikes can be low and affect the source imaging quality, substantially, Aydin et al. [15] proposed a sub-average procedure that selects a sub-set of spikes randomly (similar to bootstrapping) to improve SNR and yet preserve variability to image the underlying irritative zone using dipole fitting methods.

Our source imaging approach is different from dipole fitting as it already provides an extended solution and is robust against noise. However, we followed a similar approach and randomly drew 1,000 sets of spikes from the set of recorded spikes in 3 patients. These selection sets were unique, no two sets of the 1,000 draws were the same and were randomly generated by MATLAB. After averaging the spikes for each randomly drawn selection set, the sub-averaged spike was input into the FAST-IRES algorithm and the epileptogenic tissue was estimated for each of the 1,000 draws. The NORs were calculated for each of these 1,000 estimates and the geometric mean was calculated and compared the solution of the averaged spike (all spikes averaged; the procedure followed in this study). The results are presented in Supplementary Fig. 10 and Supplementary Table 9. It was observed that the averaged spike results were slightly exceeding the mean and median of this randomly drawn distribution, indicating that average spike analysis is not performing poorly and is not biasing our results.

The average and standard deviation of the estimated solutions for the 1,000 draws are also presented in Supplementary Fig. 10 for reference. It is observed that the average spike results are selected within the most consistent parts of the solutions, i.e. spatial locations with high average and low standard deviation. Thus, we conclude that sub-averaging proved our results to be unbiased and robust, and did not provide additional benefits, within our framework of analysis.

Supplementary Note 6: Investigating Extent Estimations in Empirical Data

The size of the estimated epileptogenic zone in all patients, using both ictal analysis and inter-ictal spike analysis, is plotted against resection size. Each dot, in the graphs depicted in Supplementary Fig. 11, indicates the data from one patient. For each patient, the averaged inter-ictal spikes were analyzed (on average patients have 29 spikes), and the area of the inter-ictal solution was calculated. Additionally, for ictal analysis, the area of each seizure analyzed in a patient was calculated and averaged to generate one data point for the patient. The results presented in Supplementary Fig. 11, indicate that the size of the estimated epileptic region from seizure and inter-ictal spike correlates significantly with the resection size. The Pearson's correlation was found to be 0.61 for ictal estimates (p-value of 0.0006) and 0.48 for spike estimates (p-value of 0.009). We further divided the patients into seizure-free and non-seizure-free (indicated by the red and blue color) and could not observe a marked difference between the extent estimation between the two groups. Pearson's correlation in effect determines how dependent two random variables are, and a correlation of 1 indicates that two variables are fully dependent on each other, which suggests that they may be driven by a common factor or variable. A correlation of 1 between estimated epileptogenic tissue and resection size, would have indicated that these processes are driven by common factors, as they would have been dependent processes. However, planning for the surgery and determining the region to be resected is not a process that is merely dependent on electrophysiological recordings and interpretation; other factors such as ictal semiology, presence of lesions observed in MRI, etc. are considered. Consequently, this process is determined by many factors and not just electrophysiological recordings, although electrophysiological recordings play an important role, undoubtedly. As a result, we did not expect our estimates to show a perfect Pearson's correlation. Our results do show positive and significantly moderate values of correlation with surgical resection, indicating that our approach provides estimates that are relevant and related to the clinical routine. It is evident that most of our estimates are smaller than the resection, which is probably due to the well-known clinical practice of making resections large enough to ensure post-surgical seizure-freedom.

There are two, outliers in Supplementary Fig. 11, that deserve more attention (a black box is used to pinpoint these two data points). First, a seizure-free case where our algorithm suggests a larger epileptogenic tissue based on ictal analysis and the other point, belongs to a patient who did not become seizure-free, yet our algorithm suggests a smaller resection area than the actual resection the patient received. In the first case, a focal lesion was observed in the MRI, and as such, the clinical team decided to only remove the lesion, while the actual seizure was originated from a larger brain tissue. For the second case, our algorithm suggested a region which was not covered by the resection (it was still in the vicinity of the resection), thus, while the overall region suggested by the algorithm was smaller it proposed areas not covered by the resection.

This agrees with the prior explanation of how clinical decisions encompass a multitude of factors, still relying heavily on electrophysiological data, as the moderate value of correlational analysis suggests. Interestingly, and in line with our observations in this paper, seizure analysis shows more correlation to clinical findings, compared to spike analysis, implying that ictal source imaging analysis might have a more positive impact in the clinical decision-making process.

Supplementary Note 7: Analysis of Variance (ANOVA) on Patient Sub-groups

We performed analysis of variance (ANOVA) for our results and different sub-populations of our patients, i.e. temporal-lobe vs extra-temporal-lobe patients, sclerosis vs. non-sclerosis patients, and patients with visible lesions in their MRI vs normal-MRI patients, without finding any significant differences between any sub-population group other than seizure-free and non-seizure-free groups. The only exception was in the spike analysis results of patients who had a clear lesion in their MRI and patients who had normal MRI. When investigated further, it was revealed that (in our database), from the 11 patients with MRI-visible lesions, 8 were among the patients who had multiple spike types in their EEG recordings. We believe that this observed difference is an indirect effect of the inconsistent spike types and not the existence of lesions.

Supplementary Tables

Table 1. Summary of Statistics (Monte Carlo Simulations)

SNR (dB)	Pearson's Correlation (between simulated and estimated extent)			Correlation between estimated and simulated time-courses of activity	
	Coefficient	95% Interval	P-value	Mean ± std	95% Interval
**20**	0.879	[0.866 0.891]	0	0.998 ± 0.0006	[0.997 0.999]
**10**	0.847	[0.830 0.862]	5.5×10^{-317}	0.997 ± 0.0012	[0.995 0.999]
**5**	0.832	[0.813 0.849]	2.7×10^{-296}	0.994 ± 0.0027	[0.988 0.999]
**0**	0.785	[0.762 0.806]	5.5×10^{-241}	0.983 ± 0.0079	[0.967 0.998]

Table 2. Summary of Simulation Results (I)

	Average Localization Error (mm)			Average Temporal Correlation		
	**5 dB**	**10 dB**	**20dB**	**5 dB**	**10 dB**	**20dB**
**1st Example**	4.1	4.1	3.6	0.96	0.97	0.97
**2nd Example**	4.3	2.8	3.5	0.96	0.96	0.97

Table 3. Summary of Simulation Results (II)

	Extent Estimation (mm) Example 1				Extent Estimation (mm) Example 2			
	**5 dB**	**10 dB**	**20dB**	**Simulated**	**5 dB**	**10 dB**	**20dB**	**Simulated**
**Source 1**	14.7	14.7	14.7	15.0	14.7	15.7	15.2	15.0
**Source 2**	10.7	10.7	10.2	8.1	7.3	7.3	7.3	8.1
**Source 3**	10.2	10.2	10.5	8.6	9.5	7.3	5.0	8.6

Table 4. Patient Outcome

Patient	Intracranial SOZ	Surgery	Outcome	Follow Up Duration	Onset (Surgery) Age	No. of Spikes Analyzed	No. of Seizures Analyzed
**1**	Right temporal grids and strips and depth electrodes on mesial side	Right anterior temporal lobectomy and amygdalohippocampectomy	ILAE-1	1 year	22 (33)	120	3
**2**	Bilateral depth electrodes into hippocampi	Right anterior temporal lobectomy and hippocampectomy	ILAE-1	3 years	22 (26)	40	2
**3**	Left frontal and temporal grids and strips and depth electrodes on mesial side	Left anterior temporal lobectomy and amygdalohippocampectomy	ILAE-1	3 years	5 months (28)	5	3
**4**	Left temporal strips and mesial depth electrodes	Left anterior temporal lobectomy and amygdalohippocampectomy	ILAE-1	32 months	25 (32)	40	3
**5**	N.A.	Left anterior frontal lobe excision	ILAE-1	30 months	17 (22)	14	3
**6**	Right temporal grids and strips and depth electrodes on mesial side	Right anterior temporal lobectomy and amygdalohippocampectomy	ILAE-1	1 year	6 (21)	21	2
**7**	Left frontal and temporal grids and strips and mesial depth electrodes	Left anterior temporal lobectomy and amygdalohippocampectomy	ILAE-2	1 year	N.A. (N.A.)	5	3
**8**	Right temporal grids and strips and depth electrodes on mesial side	Right temporal lobectomy and amygdalohippocampectomy	ILAE-1	2 years	22 (50)	xx	1
**9**	N.A.	Right temporal lobectomy and amygdalohippocampectomy	ILAE-2	2 years	11 months (46)	7	3
**10**	Right temporal grids and strips and depth electrodes on mesial side	Right temporal lobectomy and amygdalohippocampectomy	ILAE-1	1 year	N.A. (N.A.)	30	3
**11**	N.A.	Left temporal lobectomy and amygdalohippocampecomy	ILAE-1	32 months	24 (60)	9	2
**12**	Right temporal grids and strips and depth electrodes on mesial side	Left temporal lobectomy and amygdalohippocampecomy	ILAE-1	1 year	12 (53)	21	1
**13**	Right temporal grids and strips and depth electrodes on mesial side	Right temporal lobectomy and amygdalohippocampectomy	ILAE-1	1 year	12 (25)	5	4
**14**	Multiple right lateral temporal and sub-temporal grids and strips of electrodes	Extended right temporal lobectomy with amygdalohippocampectomy	ILAE-1	15 months	30 (38)	30	3
**15**	Right temporal grids and strips and depth electrodes on mesial side	Right temporal lobectomy and amygdalohippocampectomy	ILAE-1	1.5 years	1.5 (48)	25	3
**16**	Right temporal grids and strips and depth electrodes on mesial side	Right anterior temporal lobectomy and amygdalohippocampectomy	ILAE-1	1 year	41 (51)	12	3

**17**	Left frontal, parietal, temporal and occipital grids and strips of electrodes	Left parietal focal cortical resection	ILAE-1	1 year	7 (25)	80	3
**18**	N.A.	Left temporal excision; residual left temporal cavernous hemangioma	ILAE-1	20 months	17 (19)	15	--
**19**	Left frontal strips	Modified left frontal lobectomy	ILAE-1	19 months	13 (21)	20	3
**20**	N.A.	Left temporal lesion excision	ILAE-1	19 months	43 (49)	29	--
**21**	N.A.	Right temporal lesion resection	ILAE-1	20 months	25 (49)	39	3
**22**	Right frontal, orbital frontal, and bilateral interhemispheric grid/stripes/depth electrodes	Right frontal cortical resection	ILAE-1	19 months	9 (15)	16	3
**23**	Left parietal, left temporal mesial, and lateral neocortical region depth, subdural grid, and strip electrodes	Left anterior temporal lobectomy and amygdalohippocampectomy	ILAE-1	14 months	18 (23)	11	3
**24**	Bilateral temporal and frontal depth electrodes	Right mesial frontal orbital lobe excision	ILAE-2	1 year	13 (25)	8	3
**25**	Right temporoparietal and right parietal grid, right temporal depth electrodes	Right frontotemporal lobe resection	ILAE-5	1 year	21 (25)	21	1
**26**	Bitemporal depth electrodes	Right temporal lobectomy and amygdalohippocampectomy	ILAE-4	13 months	29 (39)	34	4
**27**	Right temporal strips and mesial temporal depth electrodes	Right temporal lobectomy and amygdalohippocampectomy	ILAE-3	19 months	37 (50)	68	1
**28**	Right temporal strips and mesial temporal depth electrodes	Right anterior temporal lobectomyand amygdalohippocampectomy	ILAE-4	18 months	15 months (29)	68	2
**29**	Left temporal grid and strips on the inferior temporal side and left frontal, depth electrodes on temporal and frontal	Left anterior temporal lobectomy and amygdalohippocampectomy	ILAE-3	1 year	19 (22)	29	3
**30**	Right fronto-temporal grids and strips and depth electrodes close to amygdala and hippocampus, and later right posterotemporo-parietal grids and strips	Focal temporo-occipital cortical resection	ILAE-4	1 year	14 (22)	18	3
**31**	Right temporal strips	Right temporal lobectomy and amygdalohippocampectomy	ILAE-4	13 months	2 (29)	14	4
**32**	left temporal strips and mesial depth electrodes	Left temporal lobectomy and amygdalohippocampectomy	ILAE-4	15 months	54 (58)	8	1
**33**	Left temporal strips and electrodes	Left anterior temporal lobectomy and amygdalohippocampectomy	ILAE-3	1 year	N.A. (N.A.)	61	3
**34**	Left subdural grids, strips, and depth electrodes	Lateral temporal extending into parietal cortex resection	ILAE-6	22 months	32 (34)	16	1

**35**	Right subdural grids, strips, and depth electrodes	Posterior superior margin of the sylvian fissure resection	ILAE-4	13 months	3 (37)	75	1
**36**	Left temporal subdural grids, strip electrodes, and left depth electrodes	Left anterior temporal lobectomy, amygdalohippocampectomy, and left posterior temporo-occipital corticectomy	ILAE-4	21 months	42 (47)	13	2

ILAE, International league against epilepsy; ILAE-1, completely Seizure-free no auras; ILAE-2, only auras no other seizures; ILAE-3, 1-3 seizure days per annum with/without auras; ILAE-4, 4 seizure days per annum to 50% reduction compared to baseline with/without auras; ILAE-5, 50% reduction to 100% increase in seizures compared to baseline; ILAE-6, more than 100% increase in seizures compared to baseline [18]. ILAE 1-2: seizure-free group of patients, ILAE 3-6: Non-seizure-free group of patients.

Table 5. Patient Semiology and Pathology				
**Pt. #**	**Sex**	**MRI**	**Semiology**	**Pathology**
**1**	F	Normal	Behavioral arrest, staring, chewing-type oral automatism, followed by vocalization and bimanual automatism	Mesio-temporal sclerosis, severe sub-pial and sub-cortial gliosis
**2**	M	Normal, multiple non-specific hyperintensity foci on T2 imaging and ill-defined region near the right fontal horn	No change from baseline behavior	Severe sub-corticl and sub-pial gliosis
**3**	F	Normal	Opening eyes, right hand fisting and rapid clonic movements, sitting up with occasional loud vocalization and forced head turn to right, chewing and automatism toward the end of seizure	Mild to moderate sub-pial and sub-cortical gliosis of cerebral cortex and white matter
**4**	M	Left Mesiotemporal atrophy	Loss of awareness, occasional oral automatism and left arm posturing, aphasia, post-ictal nose wiping with either left or right hand	Mesio-temporal sclerosis, severe sub-pial and sub-cortial gliosis
**5**	F	Left anterior frontal ecephalomalacia	Left hand nose wiping, mouth movements, lip smacking and swallowing, occasionally responsive and able to remember during the seizure and other times aphasic	Intra-cerebral abscess and left frontal encephalomalacia
**6**	F	Normal	Dystonic left arm posture, unresponsive and aphasic during and after seizures	Mesio-temporal sclerosis, severe sub-pial and sub-cortial gliosis
**7**	F	Normal	Aura of funny feeling in stomach, aphasia, lip smacking and swallowing	Sub-pial and sub-cortical gliosis and leptomeningeal inflammation
**8**	F	Right mesiotemporal/hippocampal atrophy	Sense of impending doom, Oral automatism, left hand tonic fisting	Mesio-temporal sclerosis, severe sub-pial and sub-cortial gliosis
**9**	F	Normal	Strange out-of-body sensation, behavioral arrest	Mesio-temporal and temporal cortex severe sub-pial and sub-cortial gliosis
**10**	F	Normal	Sudden [clonic] wave throughout the body, occasional oral automatism	hippocampal marked gliosis and sub-pial and sub-cortical temporal gliosis
**11**	F	Left Mesiotemporal atrophy	Moving both hands at onset, lip smacking, chewing	Mesial sclerosis, Severe sub-corticl and sub-pial gliosis
**12**	F	Increased T2 signal, Mild to severe general cerebral atrophy	Unresponsive, aphasic, head turning to left, left-sided clonic activity, moaning, bicycling leg movements	Mesial sclerosis, Severe sub-corticl and sub-pial gliosis
**13**	F	Normal	Oral automatism, right hand more often than left automatism, confusion	Mesial sclerosis, Severe sub-corticl and sub-pial gliosis
**14**	M	Normal	Semi-rythmic leg movements, mouth movements, rocking body side-to-side, holding left hand with right hand	Severe sub-corticl and sub-pial gliosis
**15**	M	Right hippocal atrophy and white matter loss	A Feeling of fear, moaning, breathing fast	Mild to moderate gliosis in cerebral cortex, white matter and hippocampus
**16**	F	Lesion in right infero-lateral temporal gyrus	Lip smacking and chewing, left face tightness, head turn to left, grunting	Right temporal low-grade astrocytoma, Marked gliosis in right hippocampus and sub-pial and sub-cortical gliosis in temporal cortex and white matter
**17**	F	Normal, hyperintensity on T2 imaging near right frontal horn	Right arm elevation, shaking and jerking	Severe sub-corticl and sub-pial gliosis
**18**	F	Left temporal hematoma with volume loss, and T2 hyperintensity in the left inferior temporal lobe	N.A.	left temporal mass: #1 consistent with cavernous angioma; #2 focal hemorrhage with reactive gliosis and hemosiderin
**19**	M	Encephalomalacia in the left frontal lobe, secondary atrophy of the corpus callosum	Non-forced head turn to the left, left arm moved and became immobile, right arm moved and grabbed, lip smacking, bilateral facial clonus, clonic movements maximum on the right	Cortical and white matter defect with gliosis consistent with old encephalomalacia
**20**	M	Abnormality in inferomedial left temporal lobe with stable multinodular, T2 hyperintense, and nonenhancing lesion	N.A.	Non-specific mild gliosis

**21**	M	Acavernous malformation in the right inferior temporal lobe, scattered inflammatory in the paranasal sinuses	Confused in spelling, chewing, tonic posturing of left arm associated with automatisms of the right hand, forced head deviation to the left, generalized tonic and tonic-clonic activity with postictal lethargy	Vascular malformation consistent with venous angioma with gliosis and focal hemosiderin deposition
**22**	F	Normal	Head turn to the left, eye deviation to the left with rapid breathing, moaning, sitting up from a recumbent position, mumbling	Severe sub-cortical and sub-pial gliosis
**23**	F	Normal, left hippocampus is slightly smaller than the right and increased T2 signal intensity compared to the right	Giggle and smile, confused and aphasic	Severe sub-cortical and sub-pial gliosis and mesial temporal sclerosis
**24**	M	Mild global and amygdalohippocampal volume loss	Head jerking to the left, whistle, lift up arms, touch hair, hold the person next to him, and afterwards confused	Severe sub-cortical and sub-pial gliosis
**25**	M	Subjacent brain encephalomalacic, and ex vacuo dilatation of the right lateral ventricle	Left hemiconvulsion involving the left face and arm/leg, forced left head turn	No residual arteriovenous malformation, chronic sub-pial and white matter gliosis, cortical microinfarctions
**26**	M	Normal	Brief behavioral arrest, non-forced head left turn, dystonic posturing of the left arm, lip smacking and chewing movements, nonverbal	Severe sub-cortical and sub-pial gliosis
**27**	F	Mild asymmetric decrease in volume and increased in T2 hyperintensity at the right hippocampus, nonspecific foci of T2 hyperintensities in the periventricular and subcortical white matter	Covered mouth with left hand, sat motionless with slight movements to feet, stared and swallowed, hiccupped	Mesio-temporal sclerosis, mild sub-pial and sub-cortical gliosis
**28**	M	Nonenhancing hyperintense T2 signal change at both hippocampi, with mild asymmetric decreased in volume on the left	Right hand waving, chewing, non-forced head turn to the right, left arm dystonic and left hand fisted, forced head turn to the left, ictal cry, symmetric tonic flexion of the arms and legs followed by clonic activity	Mesio-temporal sclerosis, severe sub-cortical and sub-pial gliosis
**29**	F	Normal	Acute cessation of activity staring straight ahead, tonic right shoulder abduction with or without right elbow flexion	Sub-pial and sub-cortical gliosis and leptomeningeal mixed inflammation
**30**	F	Normal	Unresponsiveness, lip smacking, head jerking to the left and then forced to the left, extension of the left upper extremity, generalized tonic-clonic seizure activity, postictal lethargy	Sub-pial and sub-cortical gliosis
**31**	M	Normal, cerebellar atrophy for age, tiny foci of abnormal T2 in the white matter of the right frontal lobe inferiorly, scattered mild to moderate mucosal thickening in the paranasal sinuses	Staring, oral automatisms, and unresponsiveness	Microdysgenesis associated with moderate gliosis and minimal glial atypica
**32**	F	Hippocampal asymmetry, suspicious for left MTS, mild cerebral and cerebellar atrophy	Non-forced head turn to the left with left upper extremity automatisms, right upper extremity, generalized with forced head turn to the right and tonic-clonic activity	Sub-pial and sub-cortical gliosis and mesio-temporal sclerosis
**33**	F	Left mesial temporal sclerosis, left hippocampus and left fornix atrophy, focal mild ectasia of the temporal horn of the lateral ventricle	Behavioral arrest, staring, turned head and body to the left, posturing of the right hand, sometimes bimanual automatisms and lip smacking, postictally amnestic and aphasic	Severe neuronal loss of gliosis in hippocampus, severe sub-cortical and sub-pial gliosis
**34**	M	Normal	Right sided hearing changes, non-rhythmic right sided facial pulling and head movements, tingling in the right leg	N.A.

**35**	M	Chronic postoperative changes of right posterior parasylvian cortical resection	Flushing, paleness, and often now a full body convulsion	Focal cortical dysplasia
**36**	M	Left mesial temporal sclerosis and postoperative changes in the temporal occipital region on the left with parenchymal loss, compensatory ventricular enlargement and a small amount of hemosiderin about the parenchymal defect	Speech difficulties	Severe cortex and white matter gliosis, sub-cortical and sub-pial gliosis, and mesial temporal sclerosis

Table 6. Summary of Statistics (Connectivity vs. Ictal Imaging Results)				
**Performance Metric**		**All Patients**	**ILAE 1-2**	**ILAE 3-6**
**Localization Error (in mm)**	**Seizure**	6 ± 5.8	5.9 ± 5.8	6.1 ± 6.3
	**Connectivity**	5.2 ± 4.92	3.9 ± 2.83	6.9 ± 6.64
**P-value**	**Perm. Test**	0.35	0.19	0.42
**Precision (normalized - 0 to 1)**	**Seizure**	0.70 ± 0.21	0.72 ± 0.23	0.66 ± 0.17
	**Connectivity**	0.73 ± 0.19	0.75 ± 0.20	0.68 ± 0.18
**P-value**	**Perm. Test**	0.34	0.36	0.4
**Recall (normalized - 0 to 1)**	**Seizure**	0.75 ± 0.19	0.76 ± 0.20	0.74 ± 0.17
	**Connectivity**	0.79 ± 0.14	0.81 ± 0.14	0.75 ± 0.14
**P-value**	**Perm. Test**	0.17	0.17	0.42
**Geometric Mean (normalized - 0 to 1)**	**Seizure**	0.70 ± 0.15	0.71 ± 0.15	0.69 ± 0.16
	**Connectivity**	0.75 ± 0.13	0.77 ± 0.13	0.70 ± 0.12
**P-value**	**Perm. Test**	0.11	0.10	0.4
**Harmonic Mean (normalized - 0 to 1)**	**Seizure**	0.68 ± 0.17	0.68 ± 0.18	0.68 ± 0.16
	**Connectivity**	0.74 ± 0.14	0.76 ± 0.15	0.69 ± 0.13
**P-value**	**Perm. Test**	0.10	0.09	0.40
**ILAE, International league against epilepsy; ILAE 1-2: seizure-free group of patients, ILAE 3-6: Non-seizure-free group of patients. Significance level is set to p < 0.05 (*). One-sided permutation test.**				

Table 7. Summary of Statistics (Spike vs. Ictal Imaging Results)				
**Performance Metric**		**All Patients**	**ILAE 1-2**	**ILAE 3-6**
**Localization Error (in mm)**	**Seizure**	6 ± 5.8	5.9 ± 5.8	6.1 ± 6.3
	**Spike**	18.1 ± 14.08	13.9 ± 11.96	23.5 ± 15.7
**P-value**	**Perm. Test**	0.0015 *****	0.0476	0.0040*****
**Precision (normalized - 0 to 1)**	**Seizure**	0.70 ± 0.21	0.72 ± 0.23	0.66 ± 0.17
	**Spike**	0.58 ± 0.38	0.65 ± 0.39	0.41 ± 0.31
**P-value**	**Perm. Test**	0.0662	0.25	0.028 *
**Recall (normalized - 0 to 1)**	**Seizure**	0.75 ± 0.19	0.76 ± 0.20	0.74 ± 0.17
	**Spike**	0.58 ± 0.32	0.61 ± 0.32	0.49 ± 0.32
**P-value**	**Perm. Test**	0.0081 *****	0.0519	0.0371 *
**Geometric Mean (normalized - 0 to 1)**	**Seizure**	0.70 ± 0.15	0.71 ± 0.15	0.69 ± 0.16
	**Spike**	0.56 ± 0.32	0.62 ± 0.32	0.44 ± 0.30
**P-value**	**Perm. Test**	0.0195 *	0.1401	0.0236 *****
**Harmonic Mean (normalized - 0 to 1)**	**Seizure**	0.68 ± 0.17	0.68 ± 0.18	0.68 ± 0.16
	**Spike**	0.55 ± 0.32	0.6 ± 0.33	0.43 ± 0.3
**P-value**	**Perm. Test**	0.0278 *****	0.1357	0.0247 *****
**ILAE, International league against epilepsy; ILAE 1-2: seizure-free group of patients, ILAE 3-6: Non-seizure-free group of patients. Significance level is set to p < 0.05 (*). One-sided permutation test.**				

Table 8. Summary of Statistics (Ictal vs. "Consistent" Spike Imaging Results)				
**Performance Metric**		**All Patients**	**ILAE 1-2**	**ILAE 3-6**
**Geometric Mean (normalized - 0 to 1)**	**Seizure**	0.70 ± 0.15	0.71 ± 0.16	0.69 ± 0.15
	**Spike**	0.63 ± 0.33	0.62 ± 0.32	0.64 ± 0.37
**Statistical Tests' Results (P-value)**	**Rank-sum Test**	0.87	0.79	0.53
	**T-Test**	0.28	0.29	0.73

***Two-sided statistical tests.**

Table 9. Statistics for Sub-averaging Results of Spikes in 3 Patients (Geometrical Mean of Precision and Recall)

Case #	Mean ± std [Median]	Average Spike Analysis	Worse? ($p < 0.05$)
**Case # 1**	0.89 ± 0.03 [0.89]	0.92	No
**Case # 2**	0.63 ± 0.18 [0.69]	0.69	No
**Case # 3**	0.79 ± 0.16 [0.84]	0.93	No

Table 10. ANOVA Analysis of Imaging Results Among Clinical Conditions

One-way ANOVA Analysis for Ictal and Inter-ictal Source Imaging Results (Geometric Mean of Precision and Recall)

	Temporal vs. Extra-temporal Lobe		Mesial Sclerosis vs. Non-sclerosis		Lesion in the MRI vs. Normal MRI		Seizure-free vs. Non-seizure-free	
	**F-statistics**	**p-value**	**F-statistics**	**p-value**	**F-statistics**	**p-value**	**F-statistics**	**p-value**
**Seizure**	2.56	0.12	0.1	0.76	2.59	0.12	0.12	0.73
**Spike**	0.2	0.66	0.26	0.61	**5.14**	**0.03 ***	1.94	0.18

4-way ANOVA Analysis for Ictal and Inter-ictal Source Imaging Results (Geometric Mean of Precision and Recall)

**Seizure**	1.29	0.27	0.05	0.82	1.86	0.19	0.01	0.93
**Spike**	0.03	0.87	0.01	0.94	**3.96**	**0.06**	1.38	0.25

Table 11. Rank-sum Statistical Test of Imaging Results for Different Clinical Conditions (Geometrical Mean of Precision and Recall)

	Temporal	Extra-temporal	p-value	Sclerosis	Non-Sclerosis	p-value	Lesion in MRI	Normal MRI	p-value
**Seizure**	0.72±0.15	0.61±0.15	0.11	0.71±0.15	0.69±0.15	0.74	0.65±0.19	0.74±0.09	0.36
**Spike**	0.57±0.34	0.5±0.29	0.3	0.52±0.39	0.58±0.29	0.89	0.43±0.33	0.68±0.27	**0.03 ***
**p-value**	0.29	0.69	xxx	0.38	0.3	xxx	0.11	0.97	xxx

***Two-sided statistical tests.**

Table 12. Number of TBF components in Each Patient for Seizure and Spike Analysis

Pt. #	No. of Spikes Analyzed	No. of Seizures Analyzed	Number of TBF components for Spike Analysis	Number of TBF components for Seizure Analysis
**1**	120	3	3	(3, 4, 3)
**2**	40	2	2	(5, 5)
**3**	5	3	4	(3, 2, 3)
**4**	40	3	6	(2, 2, 7)
**5**	14	3	3	(3, 3, 3)
**6**	21	2	3	(6, 4)
**7**	5	3	3	(4, 4, 5)
**8**	xx	1	xx	4
**9**	7	3	1	(3, 6, 3)
**10**	30	3	3	(4, 3, 3)
**11**	9	2	1	(3, 6)
**12**	21	1	2	4
**13**	5	4	2	(4, 3, 4)
**14**	30	3	5	(3, 5, 4)
**15**	25	3	4	(5, 6)
**16**	12	3	3	(4, 5, 5)
**17**	80	3	6	(6,7,4)
**18**	15	--	2 spike types – (2, 2)	--
**19**	20	3	5	(3, 4, 4)
**20**	29	--	2 spike types – (4, 2, 3)	--
**21**	39	3	2 spike types – (1, 2)	(6, 2, 4)
**22**	16	3	2 spike types – (2, 1)	(6, 4, 4)
**23**	11	3	3	(8, 6, 5)
**24**	8	3	2 spike types – (1, 1)	(4, 3, 5)
**25**	21	1	3 spike types – (2, 6, 1)	(4, 4)
**26**	34	4	2 spike types – (2, 2)	(4, 4, 5, 6)
**27**	68	1	2 spike types – (3, 2)	2
**28**	68	2	2 spike types – (3, 3)	(5, 4)
**29**	29	3	3 spike types – (2, 3, 3)	(4, 5, 4)
**30**	18	3	2 spike types – (3, 2)	(5, 6, 3)
**31**	14	4	2 spike types – (1, 2)	(4, 4, 2, 4)
**32**	8	1	3	2
**33**	61	3	2 spike types – (1, 2, 1, 4)	(5, 7, 8)
**34**	16	1	2	8
**35**	75	1	2 spike types – (3, 2)	4
**36**	13	2	5	(11, 7)

Table 13. Statistics for Correlation between Estimated Traces from FAST-IRES and Intra-cranial EEG Traces

	Anterior Temporal Region (Surface)			Para-hippocampal Region (Deep)		
**EEG/iEEG**	**Seizure #1**	**Seizure #2**	**Seizure #3**	**Seizure #1**	**Seizure #2**	**Seizure #3**
**Seizure #1**	0.63	0.57	0.64	xx	xx	xx
**Seizure #2**	0.68	0.56	0.62	0.57	0.68	0.75
**Seizure #3**	0.65	0.58	0.59	0.63	0.57	0.6
	**Mean ± std**		0.61 ± 0.04	**Mean ± std**		0.61 ± 0.07

Seizure # refers to which seizure results from the EEG recordings were compared to which seizure in the iEEG recording. In EEG Seizure #1, the deep region was not fully recovered so no value for correlation is available (xx).

Supplementary Figures

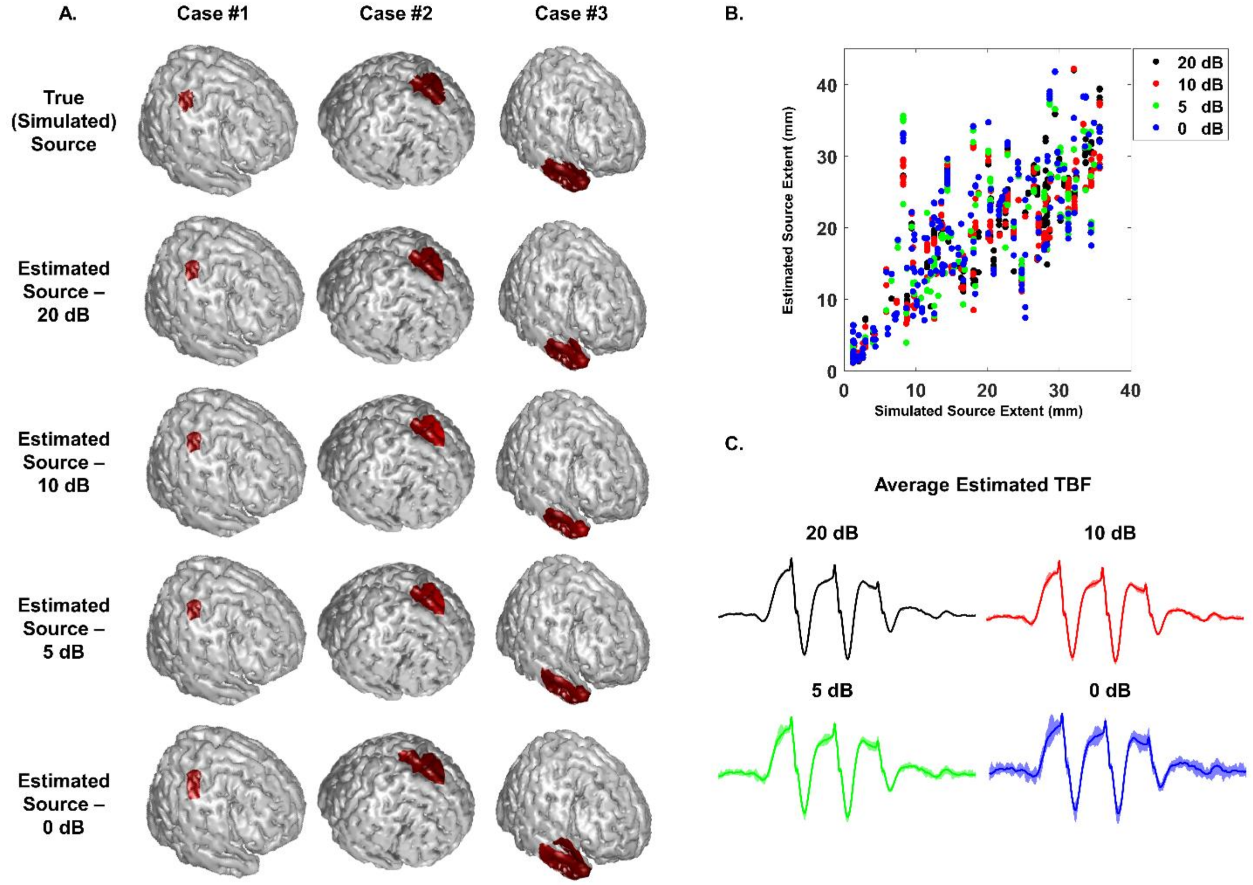

Figure 1. Monte Carlo Simulation Examples. Examples of three different sources estimated under the four tested SNRs (a). All the simulation results for the 4 different SNR conditions are plotted simultaneously for a better comparison (b). The estimated time-course of activity from all simulation examples under each SNR condition is presented in (c). The thick line is the mean among all different conditions and the shaded area shows the 99th percentile of the distribution among the 1150 simulation cases.

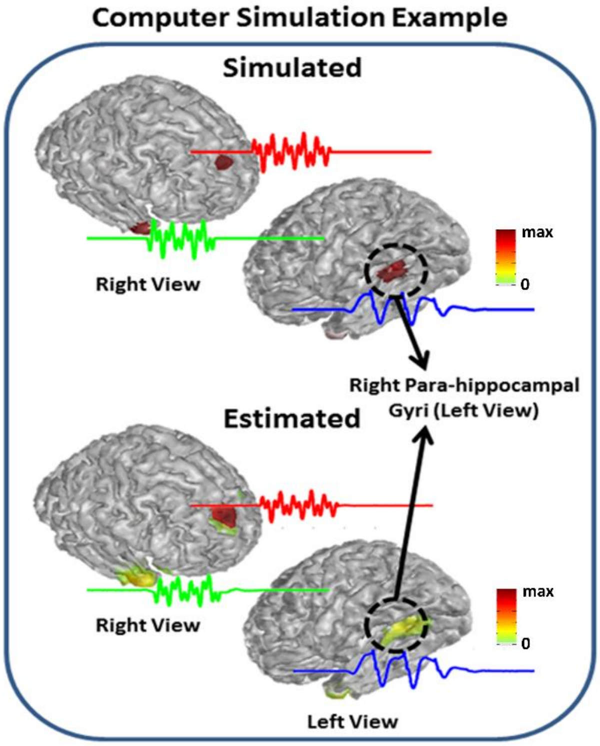

Figure 2. Simulation Results (I). The figure shows the computer simulation results where three sources with corresponding time courses were simulated and subsequently the location, extent and the time-course of activity of these sources were estimated using the FAST-IRES algorithm.

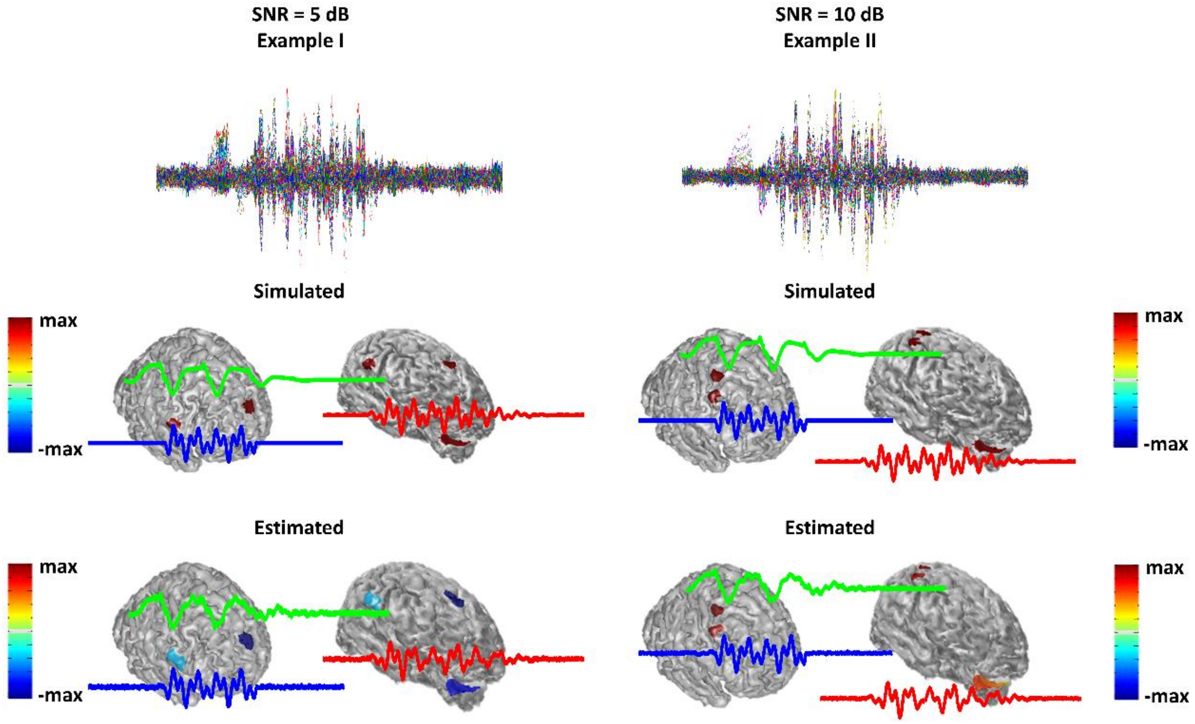

Figure 3. Simulation Results (II). The two figures show two instances of a three-source configuration simulation. (Left) the simulated and estimated sources and time-courses of a simulation scenario with a 5dB SNR and (Right) another scenario in a 10dB SNR. Complete quantitative results are reported in the supplementary Tables 2-3.

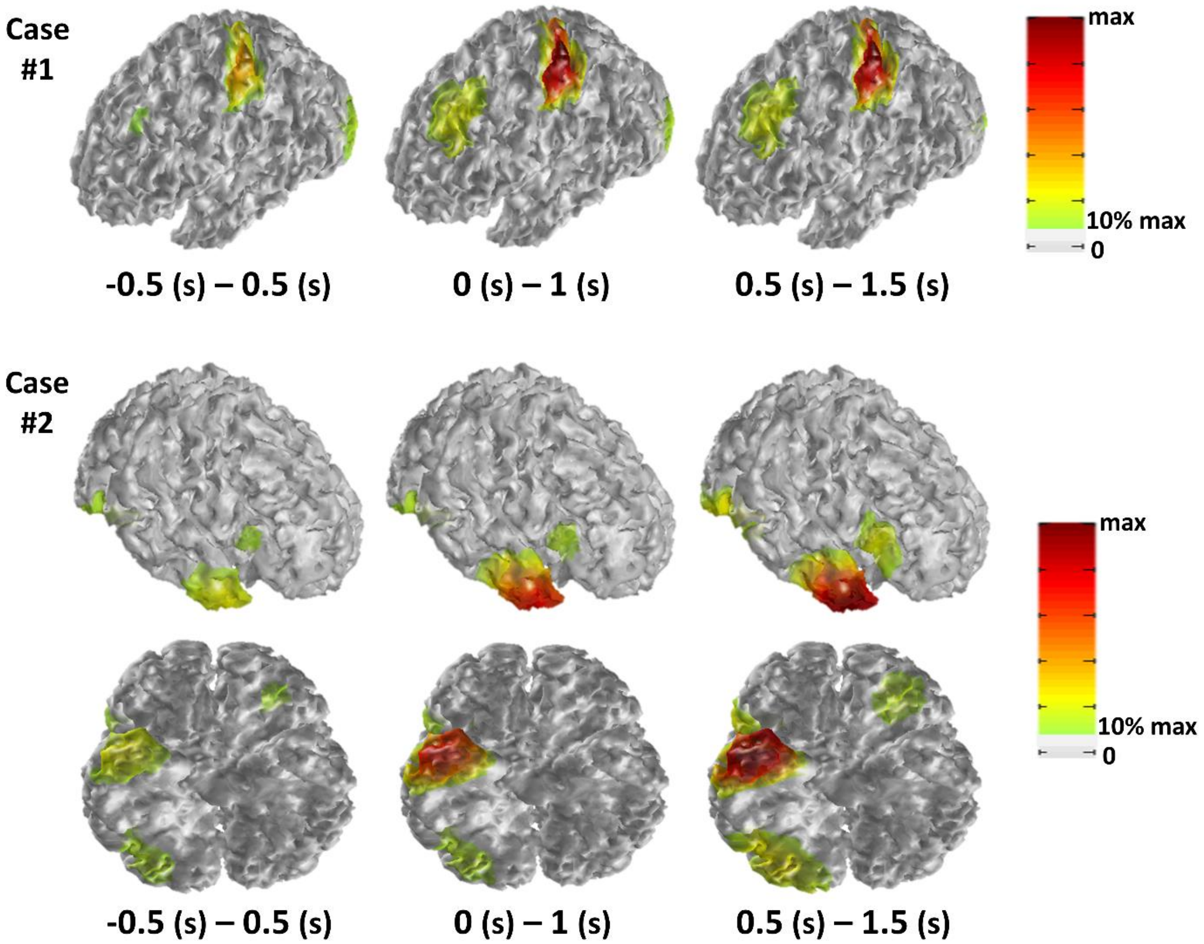

Figure 4. Motivation for Connectivity. The distribution of source energy in the narrow dominant frequency band for two of the analyzed patients during one-second intervals around seizure onset; with the passage of time, seizures propagate and might become stronger in regions other than the SOZ.

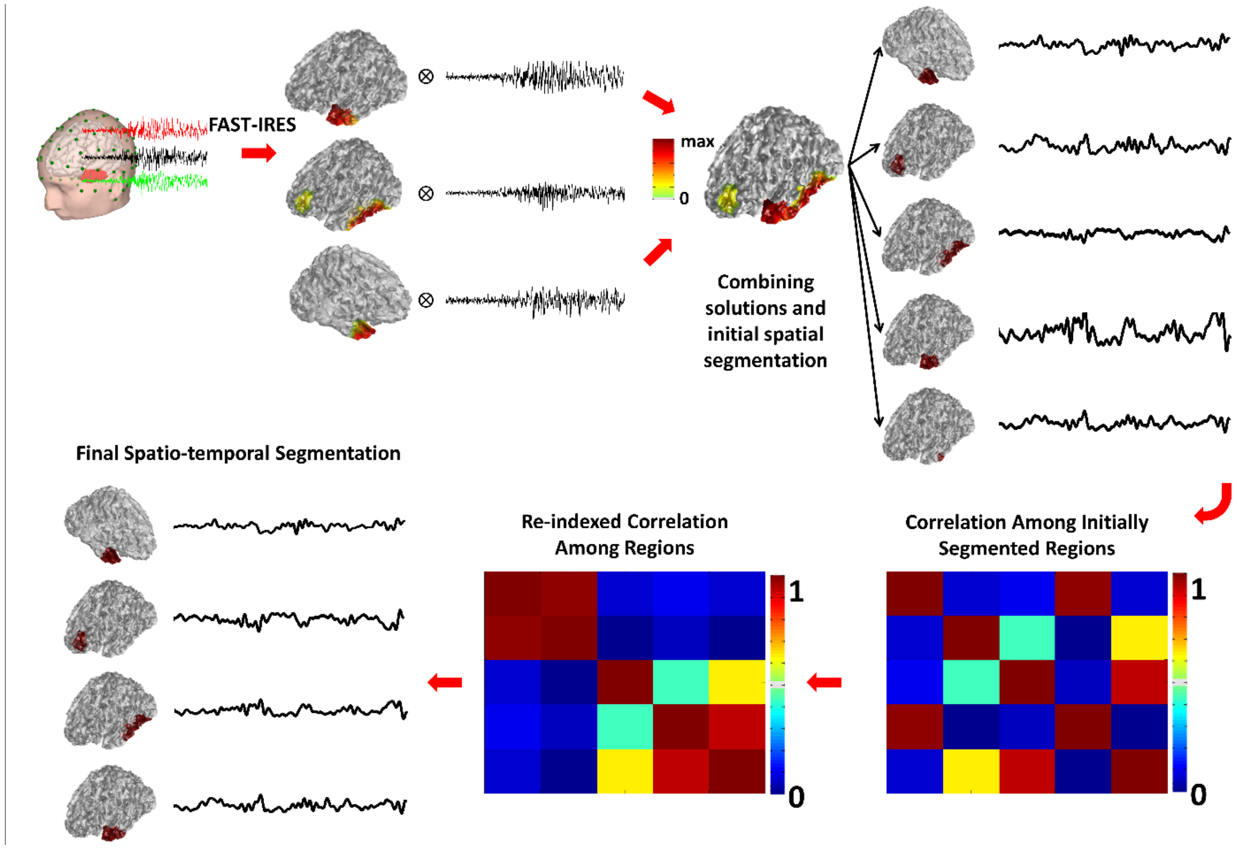

Figure 5. Forming Hyper-nodes for Connectivity Analysis. (Top Row) After the inverse problem is solved using the FAST-IRES method, regions of activity in each column of the solution are extracted. Overlapping regions are segmented as independent regions, thus a set of spatially non-overlapping segments are obtained. The average time courses from each of these regions are extracted. (Bottom Row) The correlation matrix of these extracted time-courses are formed and re-grouped to form (ideally) a block diagonal matrix. Neighboring regions are re-integrated if they demonstrate high correlation and the final non-overlapping minimally correlated segments are obtained and the average time-course of activity from these regions are extracted for further connectivity analysis.

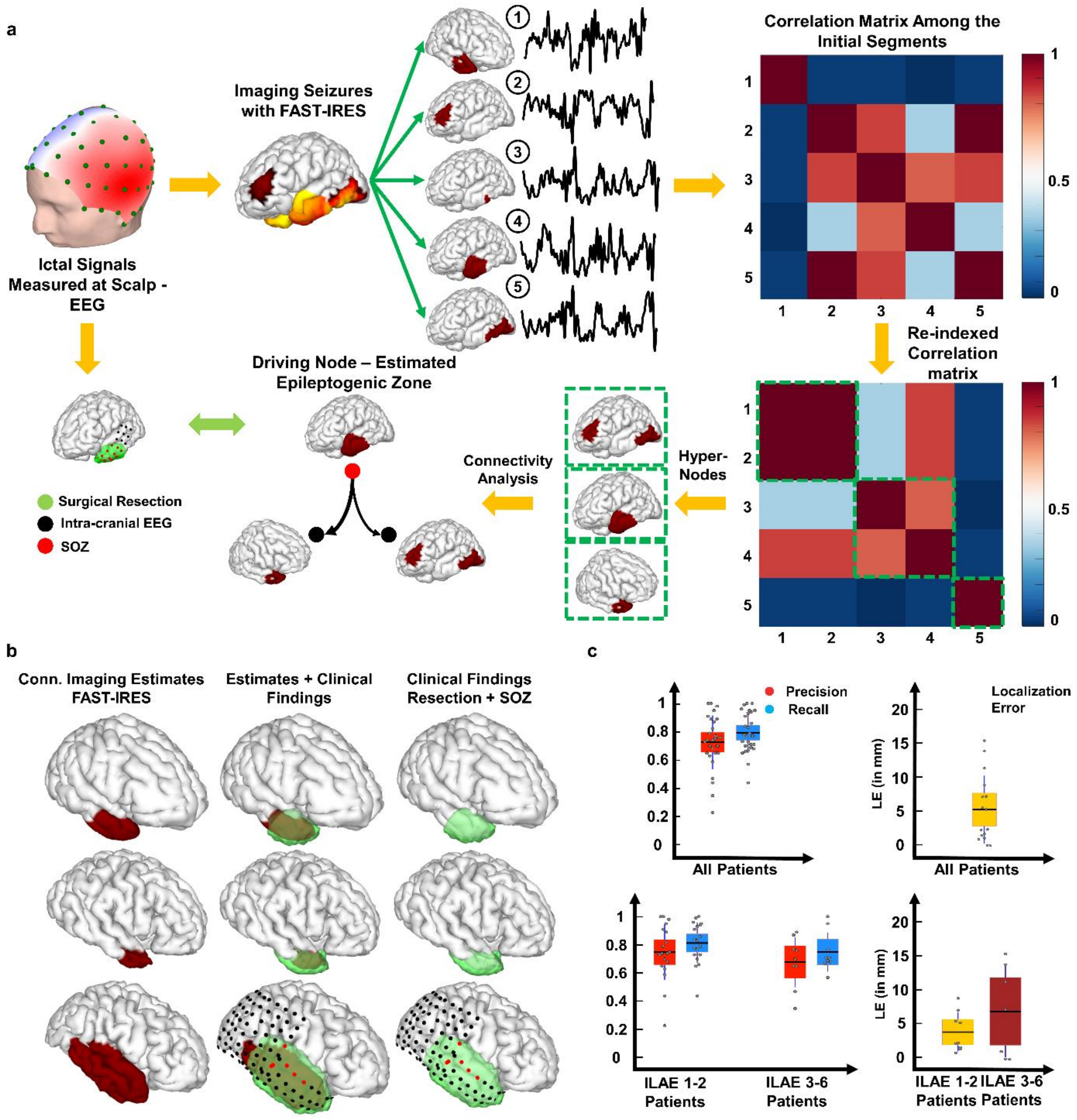

Figure 6. Connectivity Imaging Overview and Results. (a) The output of ictal imaging is a spatio-temporal distribution of source, that change over time in a fast pace, thus, regions where seizure propagated to, might be difficult to distinguish from origins of activity. FAST-IRES solutions are however, clearly distinguished from background activity. Thus, with the aid of hyper-node analysis, regions of activity are found and the region (node) driving other network nodes are determined from subsequent DTF analyses, and designated as the epileptogenic node, which can then be compared to clinical findings for validation. (b) Examples of connectivity imaging results along the clinical findings in the same patients. (c) Quantitative results of connectivity imaging results for all patients (top) and separated based on surgical outcome. Note that while the color scheme distinguishes precision vs. recall in these patients, for localization error, it is used to denote seizure-free from non-seizure-free patient groups (bottom). Each grey circle corresponds to individual patient's data. The horizontal black bar indicates the mean, the color bars

indicate the 95% confidence interval for the mean and the dark vertical bars indicate the standard deviation. To compute precision (0.73 $\pm$ 0.19) and recall (0.79 $\pm$ 0.14), 28 data points were available in total (n=28). The same analysis for seizure-free patients yielded higher precision (n=19, 0.75 $\pm$ 0.2) and recall (n=19, 0.81 $\pm$ 0.14) compared to the precision (n=9, 0.68 $\pm$ 0.18) and recall (n=9, 0.75 $\pm$ 0.14) in the non-seizure-free group. The localization error (n=16, 5.2 $\pm$ 4.92 in mm) was calculated from the data of 16 patients (n=16). The localization error (n=9, 3.9 $\pm$ 2.83 in mm) in seizure-free patients was smaller than the same value (n=7, 6.9 $\pm$ 6.64 in mm) in non-seizure-free patients. Reported values are (mean$\pm$ standard deviation). Source data are provided as a Source Data file.

a

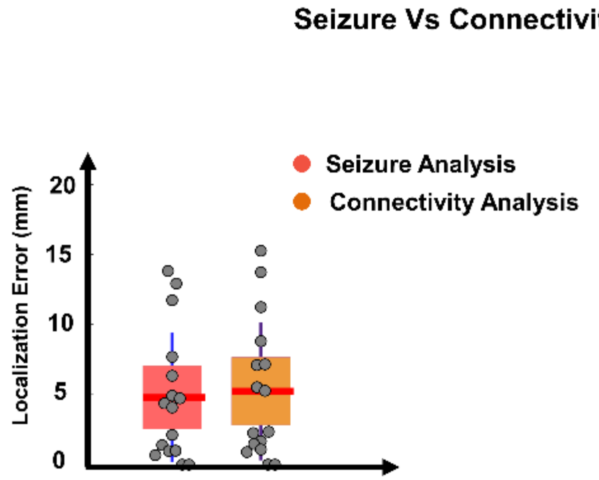

b

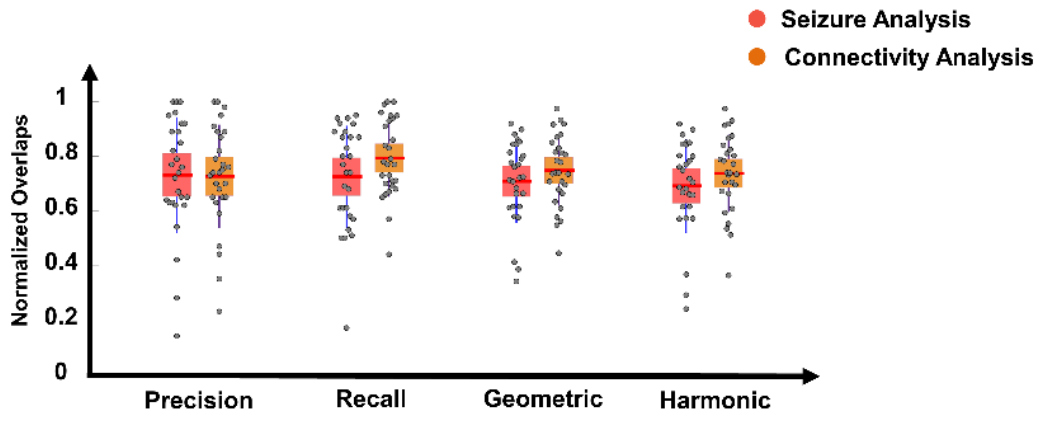

Figure 7. Ictal vs. Connectivity Imaging. (a) Localization error for connectivity and ictal imaging results (n = 16). (b) Normalized overlap ratios for ictal and connectivity imaging results (n = 28). No statistically significant difference is observed between ictal and spike imaging analysis for any metric (one-sided permutation test, refer to Supplementary Table 6 for p values). Each grey circle corresponds to individual patient's data. The red bar indicates the mean, the color bars indicate the 95% confidence interval for the mean and the dark bars indicate the standard deviation (* $p < 0.05$). Source data are provided as a Source Data file.

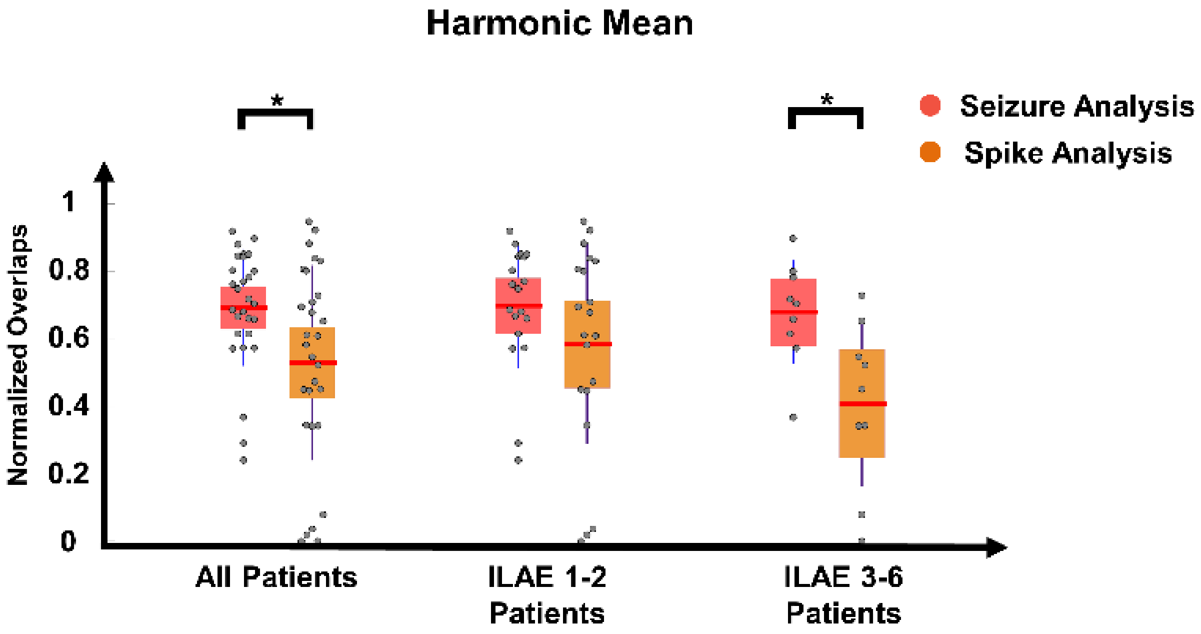

Figure 8. Ictal vs. Spike Imaging (Harmonic Mean). The Harmonic mean of spike and ictal imaging results for all patients (also broken up for seizure-free and non-seizure-free patient groups). Same results hold whether the geometric mean or harmonic mean is used (compare to Fig. 6b). The harmonic mean in all patients (n=29 for spike analysis and n=28 for ictal analysis) showed a significant difference (* $p < 0.05$) between the ictal and inter-ictal imaging results (one-sided permutation test, $p = 0.0278$). Same results were observed for non-seizure-free patients (n=9, one-sided permutation test, $p = 0.0247$), while seizure-free patients did not show such a trend (n=20 for spike analysis and n=19 for ictal analysis, one-sided permutation test, $p = 0.1357$). Each grey circle corresponds to individual patient's data. The red bar indicates the mean, the color bars indicate the 95% confidence interval for the mean and the dark bars indicate the standard deviation. Source data are provided as a Source Data file.

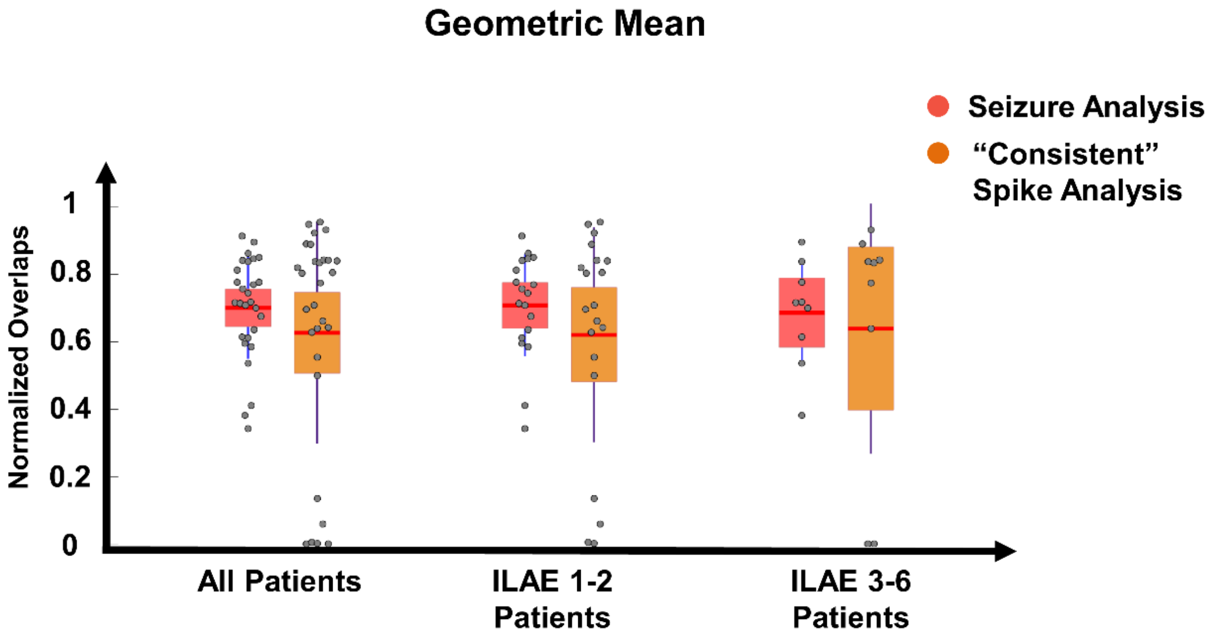

Figure 9. Geometrical Mean Differences of "Consistent" Spike and Ictal Imaging Analysis Results. No statistical differences (one-sided permutation test, refer to Supplementary Table 8 for detailed analysis) observed between "consistent" spike and seizure imaging results in total (n=29 for spike analysis and n=28 for ictal analysis), for seizure-free patient group (n=20 for spike analysis and n=19 for ictal analysis) and non-seizure-free patient group (n=9). "Consistent" spikes are defined as spikes that are ipsilateral to surgical resection. Each grey circle corresponds to individual patient's data. The red bar indicates the mean, the color bars indicate the 95% confidence interval for the mean and the dark bars indicate the standard deviation. Source data are provided as a Source Data file.

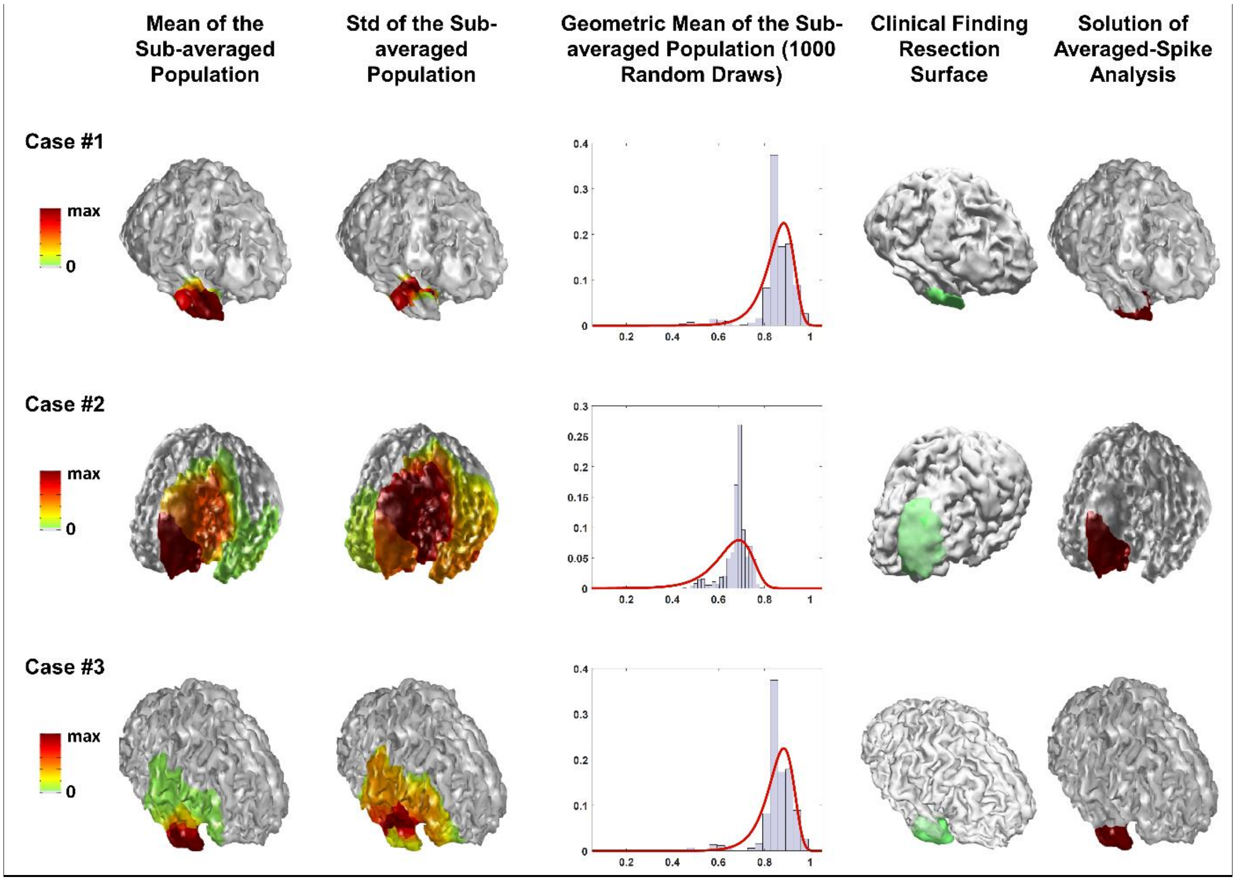

Figure 10. Sub-averaging Analysis in 3 Examples. The average and standard deviation among the 1000 solutions of the random sub-averaged spikes are presented on the left. As it can be seen on our estimates on the right, our solutions conform to the most consistent part of these distribution, i.e. where average results are high in amplitude and standard deviation is low. The histograms depict the probability distribution of the geometrical means of these 1000 draws (for quantitative results kindly refer to Supplementary Table 9). Source data are provided as a Source Data file.

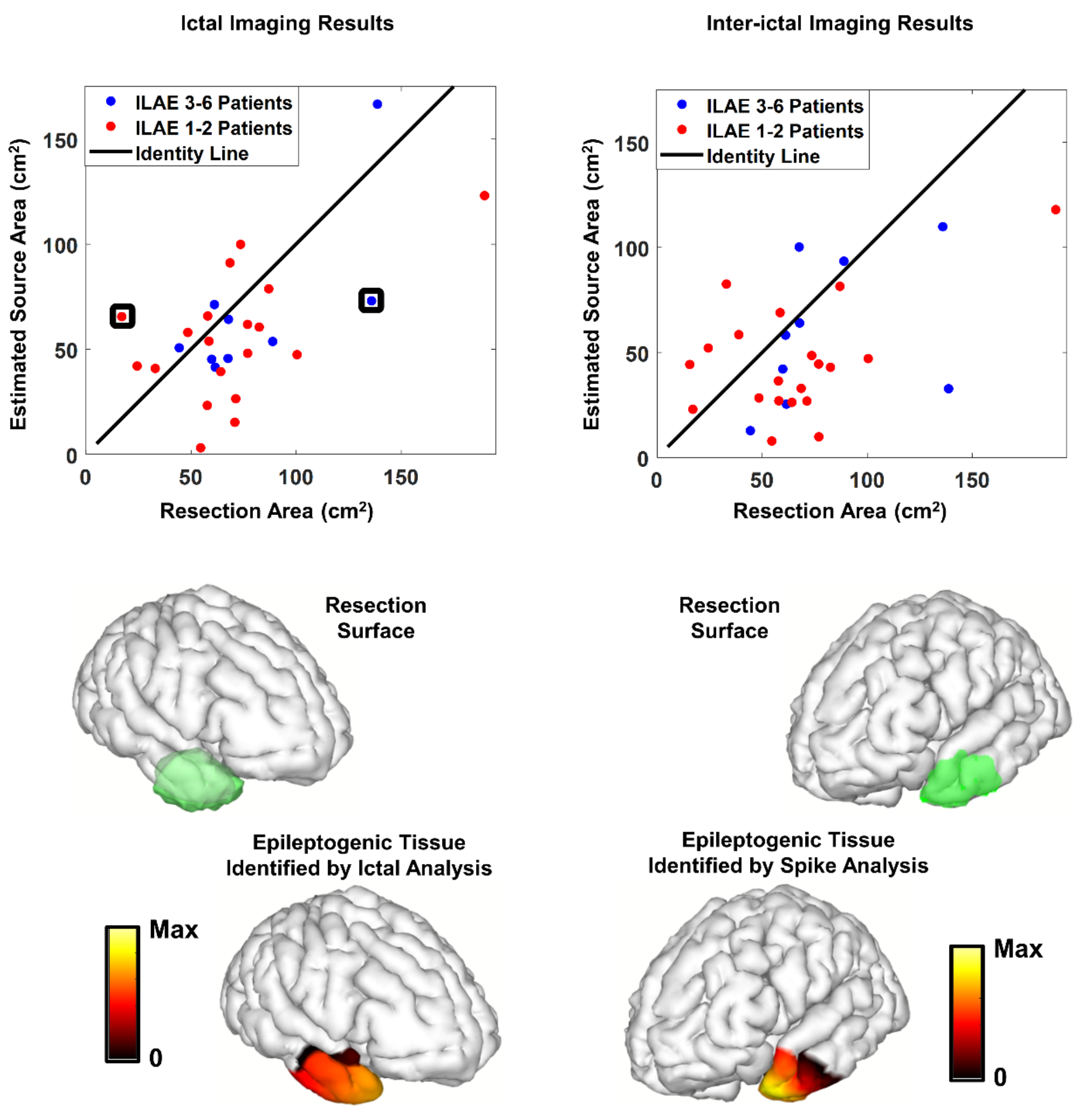

Figure 11. Estimated and "True" Extent of Empirical Data. The area of resection and estimated sources' area (for both inter-ictal and seizure analysis) are plotted against each other. Two examples of estimated epileptogenic tissue from ictal and inter-ictal imaging are provided as well. Source data are provided as a Source Data file.

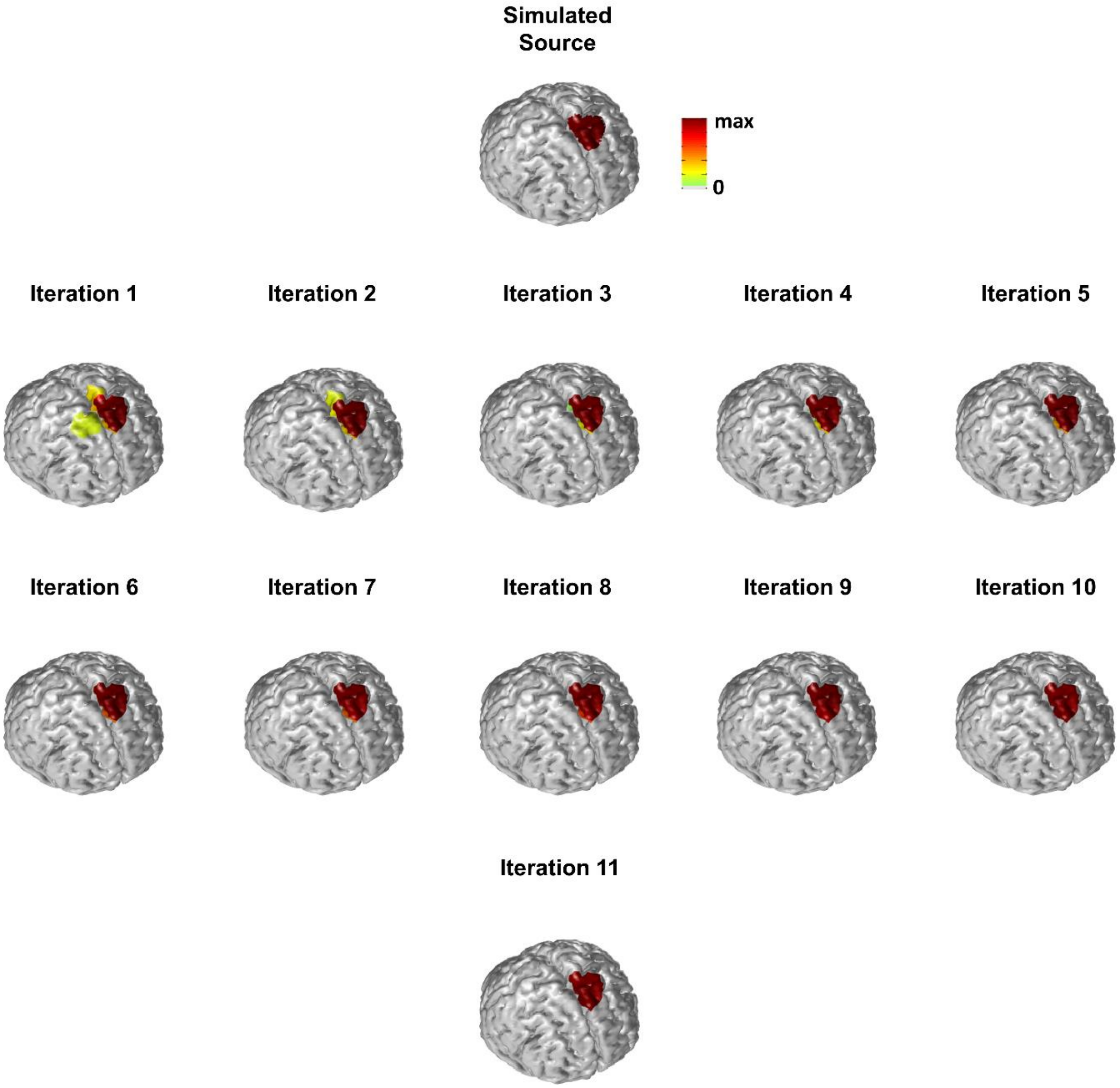

Figure 12. Effect of Iterations on Estimations. While the iterative re-weighting scheme is key in distinguishing signal from background noise in the FAST-IRES framework, it usually converges within a few iterations. Continuing iterations will not shrink the solution to zero or an overly focused source.

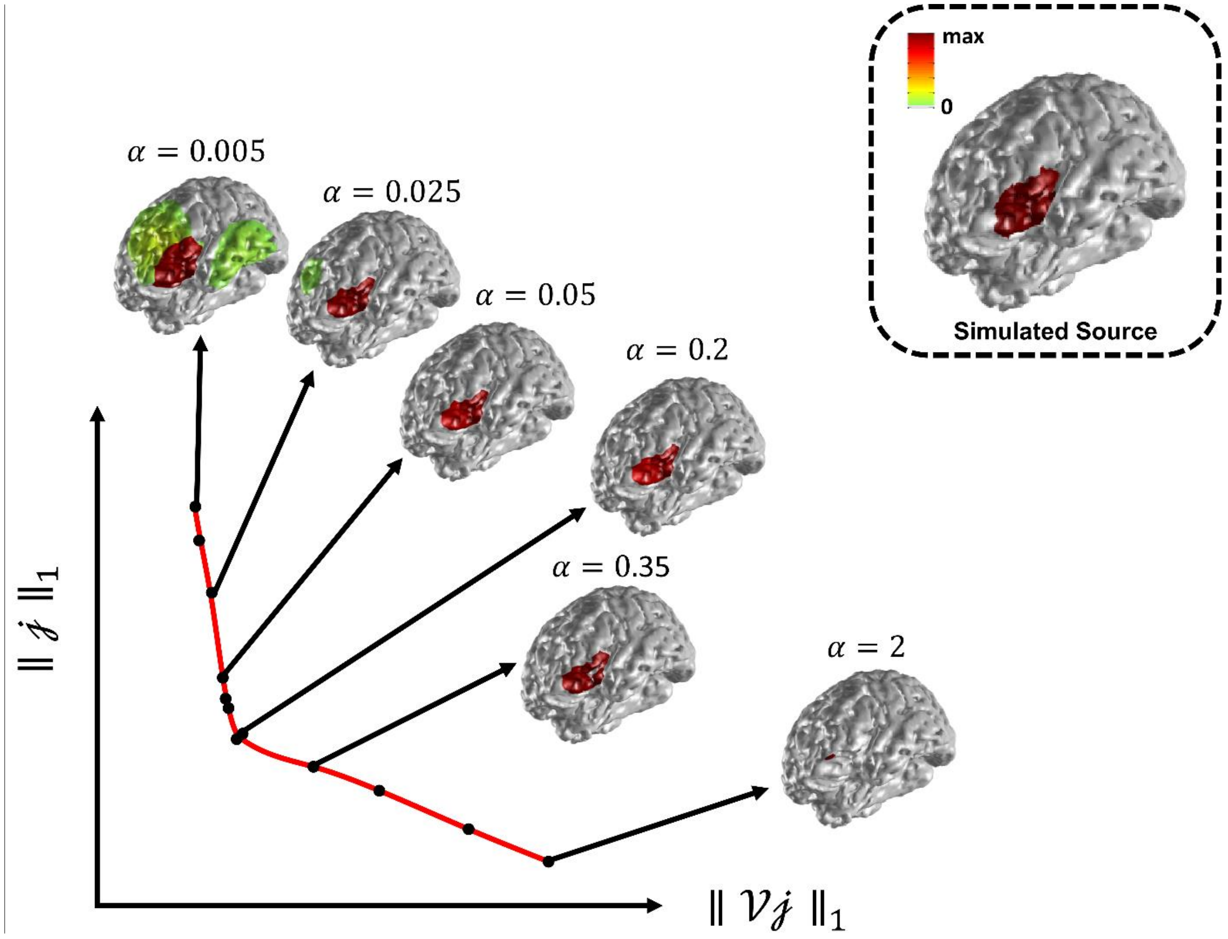

Figure 13. Example of an L-curve and Choice of α on Estimations. The L-curve helps us choose the α parameter that balances between the two terms of the optimization problem, in a systematic manner. While, choice of α matters in determining the source extent, as the example provides, a reasonable range of values are possible and will still yield excellent solutions.

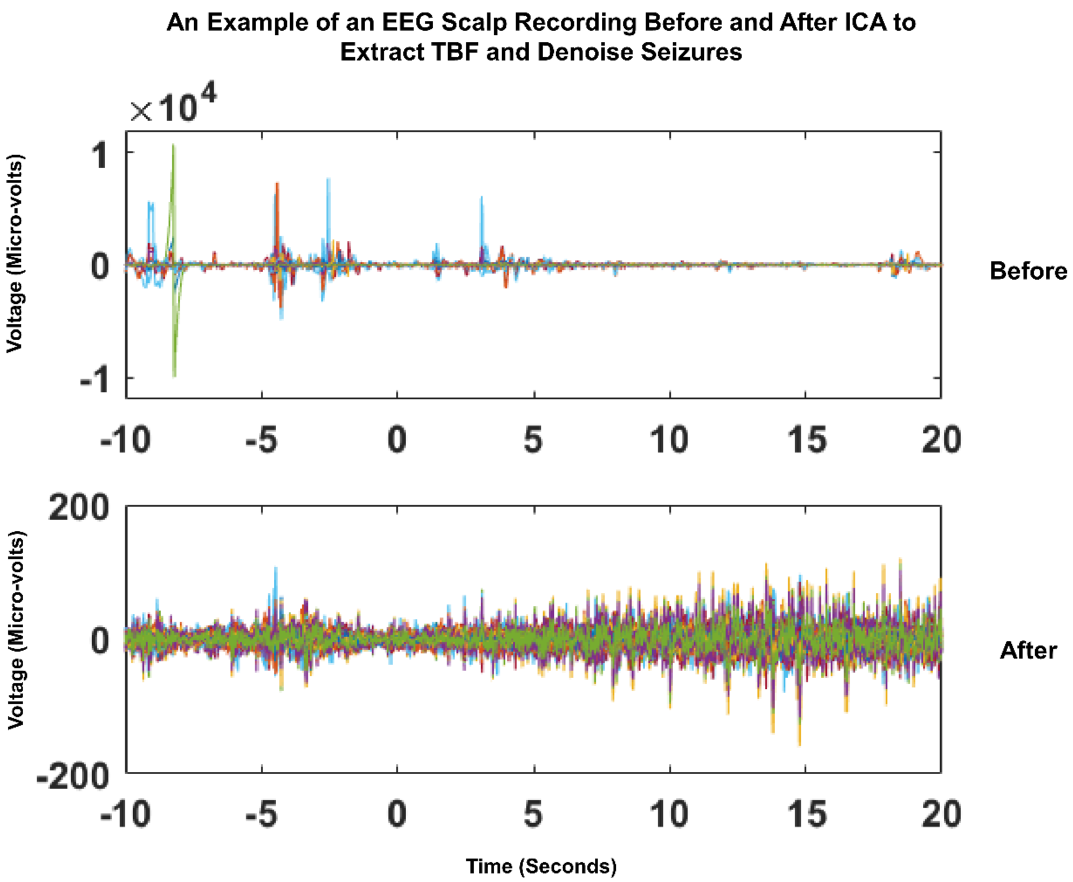

Figure 14. Denoising Effect of ICA. A typical example of how ICA can clean up EEG ictal recordings. Due to movement, eye rolling, eye blinks and many other artefacts, ictal recordings are not easy to process in their raw form and source imaging based on raw data may not be accurate enough, so, a careful pre-processing is recommended.

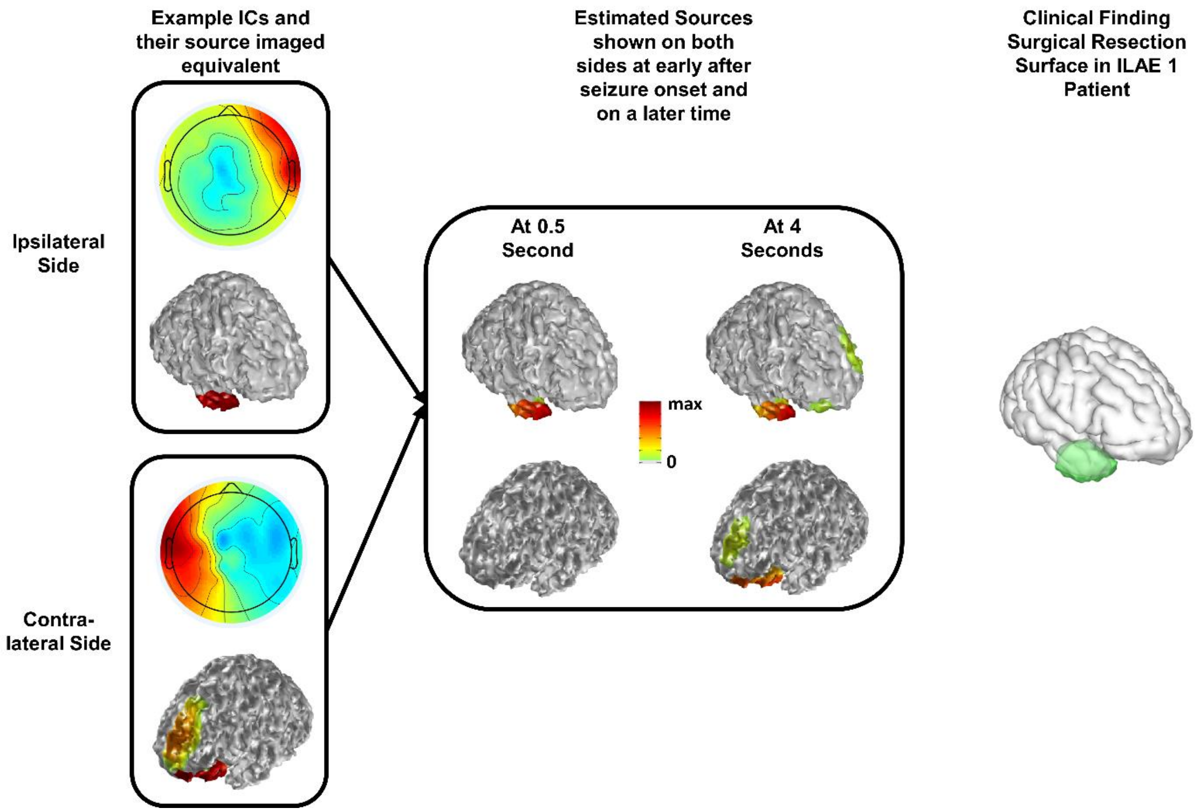

Figure 15. An Example of Ictal Propagation in a Temporal Lobe Epilepsy Case. In this example we can clearly see that ICA determined components on both hemispheres (imaged components in source space correspond to the time-course of activity of the presented spatial components, i.e. the TBF elements). As time passes the ictal activity can propagate to the contra-lateral side as quick as a few seconds after seizure onset.

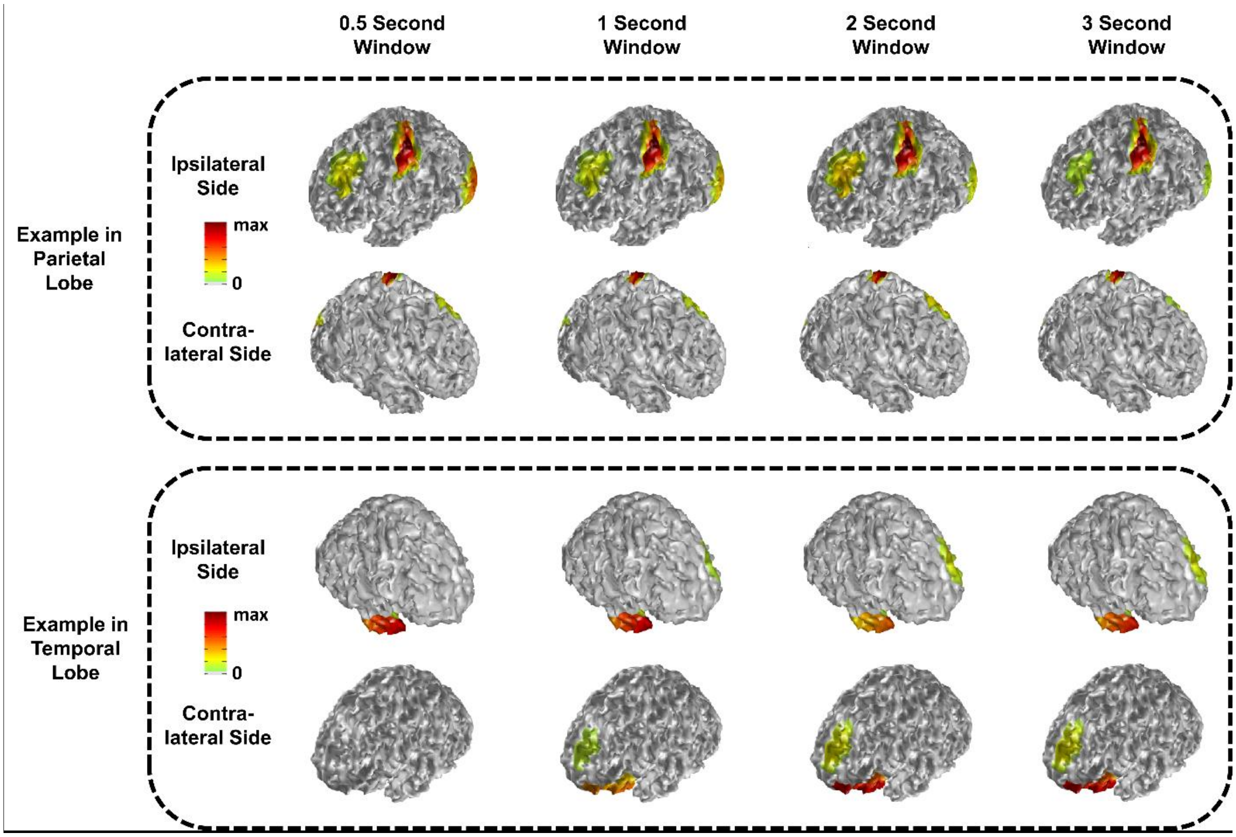

Figure 16. Effect of Window Size on Epileptogenic Zone Estimations. Two cases are presented in this figure, a parietal-lobe case on the top row and temporal-lobe on the bottom row. For each case, the ipsilateral and contralateral view are presented to provide a view of how much seizure activity may or may not propagate following seizure onset. After a few seconds following the seizure onset, ictal activity may propagate to other brain regions, thus the window of analysis, i.e. the average activity of the dominant ictal frequency, cannot be too long as indicated by the temporal-lobe case presented here.

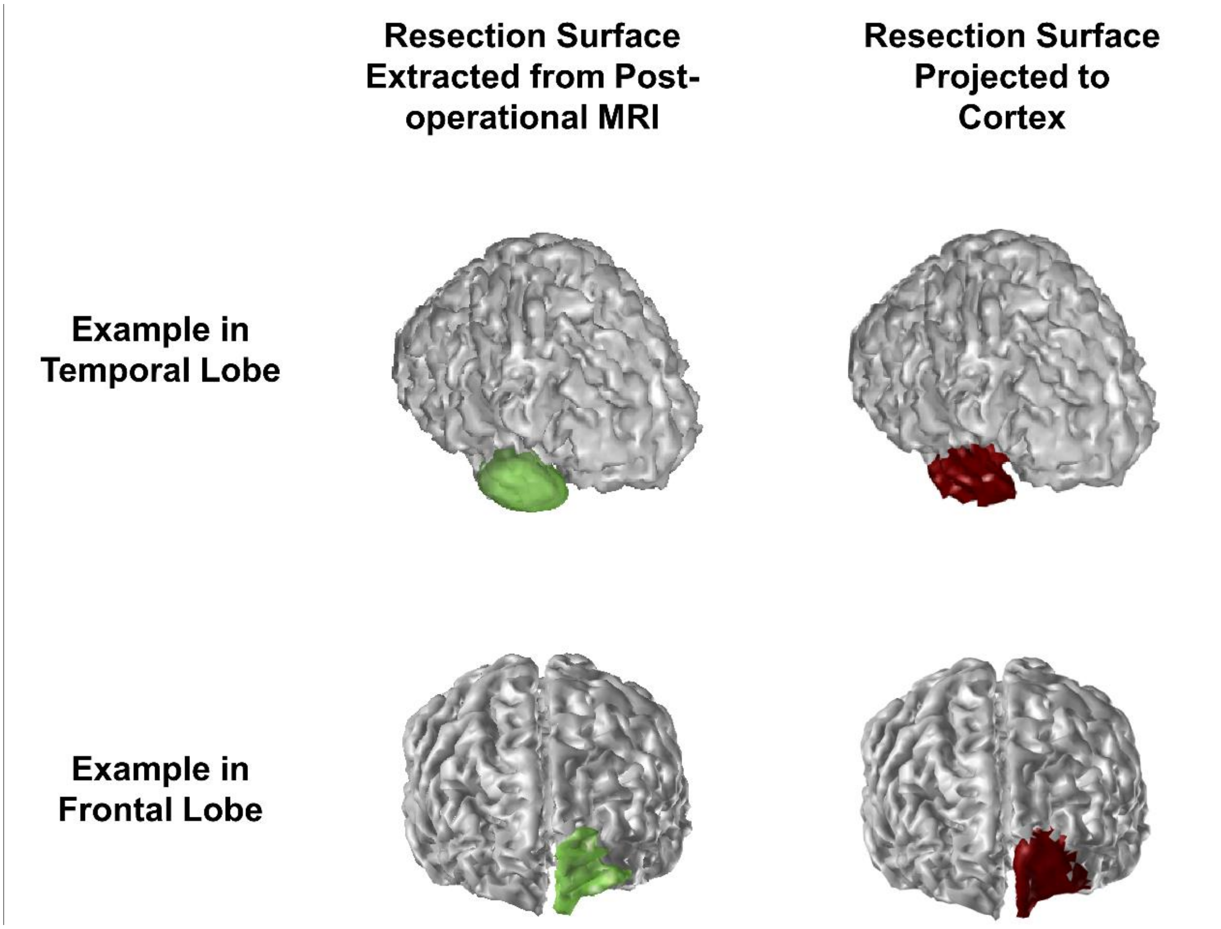

Figure 17. Example of Projecting Resection Surface to Cortical Surface. Surgical volume surfaces extracted from post-surgical MRI (co-registered to pre-surgical MRI) were carefully projected to the cortical surface, where our solution space is located, so that subsequent analyses (calculating precision and recall) could be performed.

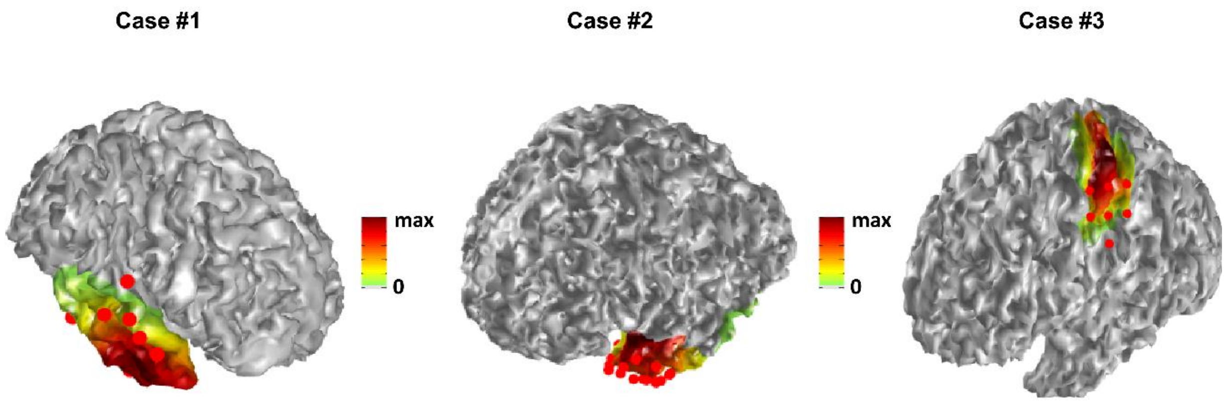

Figure 18. SOZ Electrodes and Estimated Solutions Relative Positioning. In three typical examples we are showing how SOZ electrodes cover our estimated SOZs and are close to the maximum activity (our solution due to the edge-sparsity enforcement in FAST-IRES is quite uniform).

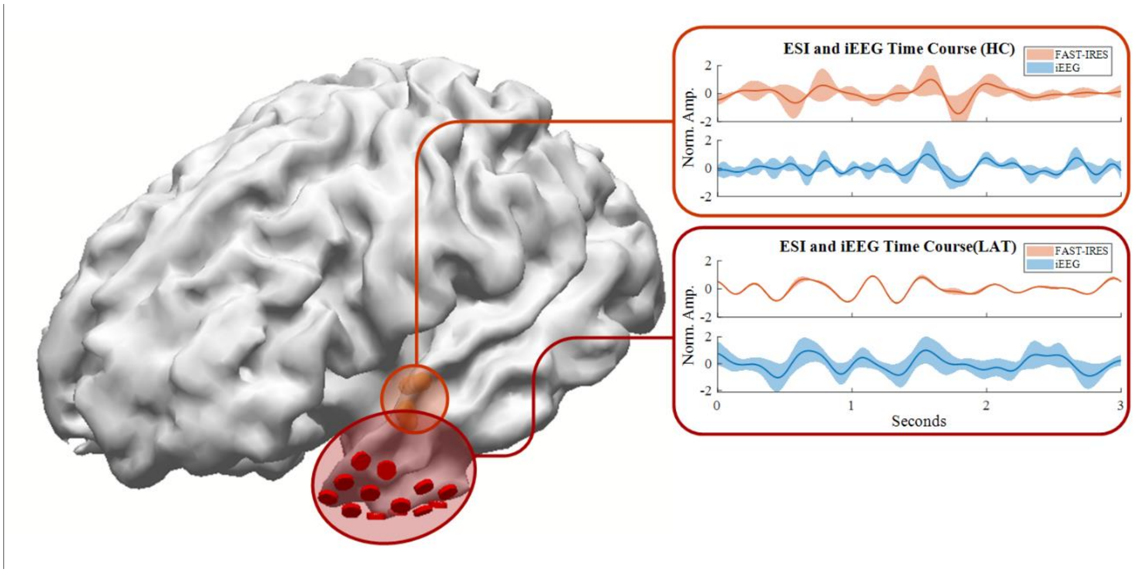

Figure 19. Comparing Estimated Time-courses from EEG to Intra-cranial Recordings. In this temporal lobe epilepsy patient, where deep mesial electrodes were implanted, the estimated time-courses from FAST-IRES solution near the SOZ electrodes were averaged and compared to the intra-cranial traces. We looked at anterior regions and deep, near the hippocampus and para-hippocampal cortex regions, separately as the signal looks quite different and found a high correlation between these traces as indicated in the right panel. For the two regions, i.e. deep mesial cortical electrodes near the hippocampus (HC) and the antero-lateral temporal (LAT) surface electrodes, the intra-cranial recorded traces, in blue, are compared to the estimated time-course of activity from FAST-IRES estimates, in red.

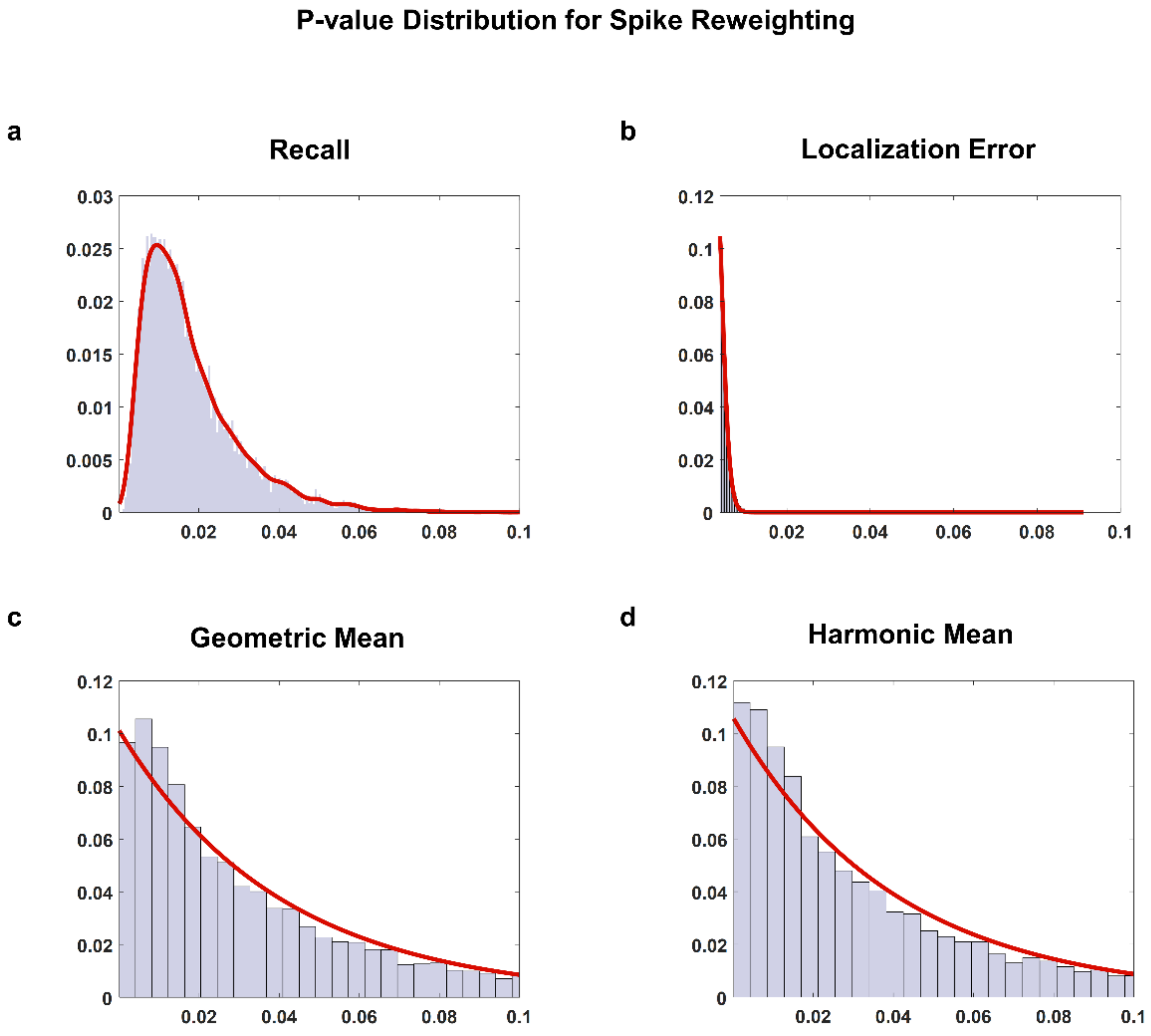

Figure 20. Weighting Strategy for Spike Data Analysis. P-value probability distributions for performance measures where statistically significant difference between spike and seizure imaging results is observed, when the weighting is randomly assigned for different spike types. In all presented cases, over 74% of the p-values are less than the significance level of 0.05, showing the independence of the obtained results to the employed weighting strategy. Source data are provided as a Source Data file.

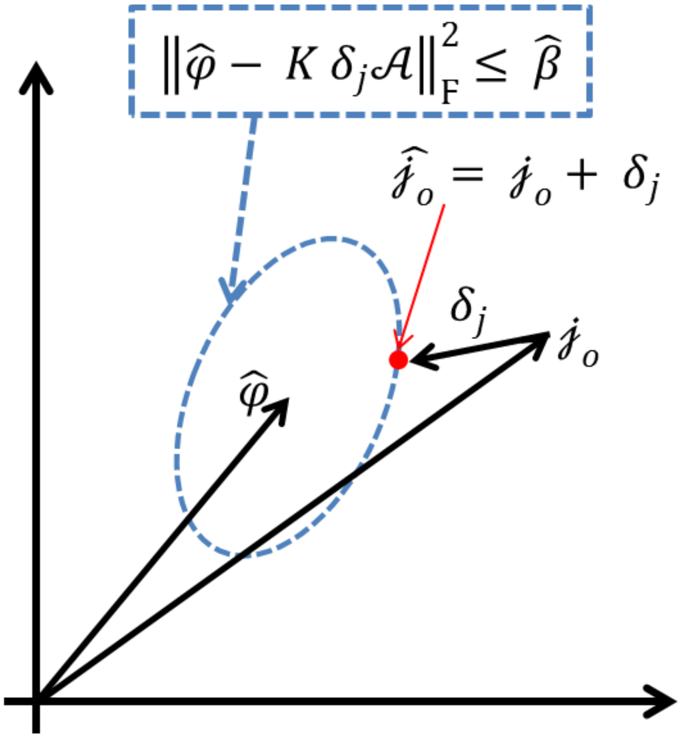

Figure 21. Projection to Hyper-ellipsoid. In this figure we can schematically observe the role of the constraint and how hyper-ellipsoid surface projection works.

Supplementary Methods.

Efficient Algorithm for Implementing FAST-IRES

An efficient algorithm will be detailed here which is used to implement IRES in a manner suitable for tracking dynamic brain signals with fast variations over time. In order to solve the problem efficiently, it is assumed that extended sources with coherent activity over the extended source patch are generating the EEG or MEG signal, in a given time window.

Based on an idea from the convex optimization literature, namely the fast iterative shrinkage-thresholding algorithm (**FISTA**) [19], we have proposed a fast spatio-temporal IRES (**FAST-IRES**) algorithm. First the time basis function is estimated from surface measurements such as EEG and/or MEG (as outlined in Supplementary Note 1), then the underlying extended source generating these signals will be estimated. The mathematical formulation is presented in Equation (2).

Previously we have discussed how the iterations improve our estimates at every step and how to update the weights at every iteration, how to find β^2 and determine α and how to determine $\mathbf{A}$ using component analysis or time-frequency methods such as wavelet analysis [4]; what we will discuss here is how to solve the basic problem of the following form which is the backbone for solving the FAST-IRES problem:

$$\hat{\mathbf{j}} = \underset{\mathbf{j}}{argmin} \parallel \mathbf{W}_d \odot (\mathbf{Vj}) \parallel_1 + \ \alpha \parallel \mathbf{W} \odot \mathbf{j} \parallel_1$$

$$subject\ to \quad \|\boldsymbol{\phi}(\mathrm{t}) - \ \mathbf{KjA}\|_{\mathcal{F}}^2 \ \leq \ \beta^2 \tag{5}$$

In this formulation we have concatenated all the $\mathbf{W}_{d,i}$ and $\mathbf{W}_i$ into matrices of the same size as $\mathbf{Vj}$ and $\mathbf{j}$ respectively; noting that the weight matrices were diagonal matrices, they can be narrowed down to column vectors without loss of data (diagonal elements of the matrix) and subsequently concatenated

together to form a matrix of corresponding sizes to $\mathbf{Vj}$ and $\mathbf{j}$. The operator $\odot$ denotes an element-by-element matrix multiplication.

Algorithms for solving convex optimization problems which impose sparsity on total variation domain, have been proposed previously, but such algorithms are very complicated [20]. Our idea is to implement this problem using augmented Lagrangian methods [21]; that is, to separate variables relating to the current density $\mathbf{j}$ and edge variables $\mathbf{Vj}$, in order to solve the problem in Eq. 5 efficiently using block-coordinate descent algorithms. We have first re-written the problem in Eq. 5 in its equivalent following form:

$$(\hat{\mathbf{j}}, \hat{\mathbf{Y}}) = \underset{(\mathbf{j},\mathbf{Y})}{argmin} \parallel \mathbf{W}_d \odot \mathbf{Y} \parallel_1 + \alpha \parallel \mathbf{W} \odot \mathbf{j} \parallel_1$$

$$subject\ to \quad \|\boldsymbol{\Phi}(\mathrm{t}) - \mathbf{KjA}\|_{\mathcal{F}}^2 \leq \beta^2$$

$$\mathbf{Y} = \mathbf{Vj} \tag{6}$$

And then using the augmented Lagrangian idea, solve the following modified problem which is not only equivalent to solving Eq. 6 but also a separable and strictly convex optimization problem [21]; which can be solved extremely efficiently using existing convex optimization algorithms such as FISTA:

$$(\hat{\mathbf{j}}, \hat{\mathbf{Y}}) = \underset{(\mathbf{j},\mathbf{Y})}{argmin} \parallel \mathbf{W}_d \odot \mathbf{Y} \parallel_1 + \alpha \parallel \mathbf{W} \odot \mathbf{j} \parallel_1 + \frac{\lambda}{2} \|\mathbf{Y} - \mathbf{Vj}\|_{\mathcal{F}}^2$$

$$subject\ to \quad \|\boldsymbol{\Phi}(\mathrm{t}) - \mathbf{KjA}\|_{\mathcal{F}}^2 \leq \beta^2$$

$$\mathbf{Y} = \mathbf{Vj} \tag{7}$$

Where, λ is a smoothing hyper-parameter which can be tuned from data easily. In order to solve the problem in Eq. 7, we will follow a block coordinate approach where $\mathbf{j}$ is updated (estimated) first, assuming that $\mathbf{Y}$ is given, then once $\mathbf{j}$ is estimated (updated) $\mathbf{Y}$ will be updated based on the recently estimated $\mathbf{j}$, at the next iteration. This alternation will continue until the solution converges to a fixed point,

i.e. $\mathbf{j}$ and **Y** do not vary much at successive iterations and converge to a solution. It is proven in convex optimization theory that such a strictly convex optimization problem under relatively achievable mathematical conditions will converge to the optimal solution regardless of initiation [19,21]. Basically, the problem proposed in Eq. 7 will be solved in the following manner; by solving the following sub-problems:

Problem S1:

$$\mathbf{j}^{K+1} = \underset{\mathbf{j}}{argmin}\ \alpha \parallel \mathbf{W} \odot \mathbf{j} \parallel_1 + \frac{\lambda}{2} ||\mathbf{Y}^K - \mathbf{Vj}||_{\mathcal{F}}^2 + (\mathbf{u}^K)^T(\mathbf{Y}^K - \mathbf{Vj})$$

$$subject\ to \quad ||\boldsymbol{\phi}(\mathrm{t}) - \mathbf{KjA}||_{\mathcal{F}}^2 \leq \beta^2 \tag{8}$$

Once $\mathbf{j}$ is updated then **Y** has to be updated based on the updated value, as outlined in problem S2:

Problem S2:

$$\mathbf{Y}^{K+1} = \underset{\mathbf{Y}}{argmin} \parallel \mathbf{W}_d \odot \mathbf{Y} \parallel_1 + \frac{\lambda}{2} ||\mathbf{Y} - \mathbf{Vj}^{K+1}||_{\mathrm{F}}^2 + (\mathbf{u}^K)^T(\mathbf{Y} - \mathbf{Vj}^{K+1})$$

$$subject\ to \quad ||\boldsymbol{\phi}(\mathrm{t}) - \mathbf{Kj}^{K+1}\mathbf{A}||_{\mathcal{F}}^2 \leq \beta^2 \tag{9}$$

But given that the condition $||\boldsymbol{\phi}(\mathrm{t}) - \mathbf{Kj}^{K+1}\mathbf{A}||_{\mathcal{F}}^2 \leq \beta^2$ is already satisfied in the previous succession in Eq. (8) in solving problem **S1** (and that we are not updating **j)**, problem **S2** is really an unconstrained optimization problem as follows:

Problem S2:

$$\mathbf{Y}^{K+1} = \underset{\mathbf{Y}}{argmin} \parallel \mathbf{W}_d \odot \mathbf{Y} \parallel_1 + \frac{\lambda}{2} ||\mathbf{Y} - \mathbf{Vj}^{K+1}||_{\mathcal{F}}^2 + (\mathbf{u}^K)^T(\mathbf{Y} - \mathbf{Vj}^{K+1}) \tag{10}$$

Update U:

$$\mathbf{u}^{K+1} = \mathbf{u}^K + (\mathbf{Y}^{K+1} - \mathbf{Vj}^{K+1}) \tag{11}$$

This step is the dual ascent update on the dual variable u, which is necessary to guarantee the constraint, $\mathbf{Y} = \mathbf{Vj}$. This update must follow every succession at which problems **S1** and **S2** are solved.

Thus, sub-problems **S1** and **S2** and updating **U** will have to be solved in succession to solve our problem proposed in Eq. 5. Solving the sub-problems **S1** and **S2** is easy and we have adopted a modified FISTA algorithm to solve these sub-problems. The modification is in implementing the hyper-ellipsoid constraint of our problem or the noise constraint, $\|\boldsymbol{\phi}(\mathrm{t}) - \mathbf{KjA}\|_{\mathcal{F}}^2 \leq \beta^2$. This constraint basically states that any solution obtained for problems **S1** and **S2** that minimizes the goal functions, must also satisfy the constraint; geometrically speaking the solution must fall within this hyper-ellipsoid. This means that any solutions obtained that minimizes the goal functions of **S1** must then be projected to the boundary (hyper-surface) of this hyper-ellipsoid. This means that for a given $\mathbf{j}_o$ that say minimizes problem **S1**, we will have to find the correction vector δ_j such that $\widehat{\mathbf{j}_o} = \mathcal{P}_{\widehat{\beta}}(\mathbf{j}_o) = \mathbf{j}_o + \boldsymbol{\delta}_j$ falls within the hyper-ellipsoid ($\mathcal{P}_{\widehat{\beta}}(\,)$ denotes the projection operator). Mathematically speaking the projection problem can be described (formulated) as follows:

Problem P:

$$\widehat{\boldsymbol{\delta}_j} = \underset{\boldsymbol{\delta}_j}{argmin} \ \left\|\boldsymbol{\delta}_j\mathbf{A}\right\|_{\mathcal{F}}^2$$

$$subject\ to \quad \left\|\widehat{\boldsymbol{\phi}} - \mathbf{K}\boldsymbol{\delta}_j\mathbf{A}\right\|_{\mathcal{F}}^2 \leq \hat{\beta}$$

This is schematically shown in Supplementary Fig. 21 (note that, $\widehat{\boldsymbol{\phi}} = \boldsymbol{\phi}(\mathrm{t}) - \mathbf{Kj}_o\mathbf{A}$). The closed form solution to problem **P** is as follows:

Problem P:

$$\widehat{\boldsymbol{\delta}_j} = \lambda^*\mathbf{K}^T(\mathbf{I} + \lambda^*\mathbf{KK}^T)^{-1}\widehat{\boldsymbol{\phi}}\mathbf{A}^T(\mathbf{AA}^T)^{-1} \tag{12}$$

Where λ^* is a scalar which needs to be calculated by solving the following problem (which, can be solved numerically using Newton's method pretty easily):

$$\sum_{i=1}^{E} \frac{\sum_t \boldsymbol{\varphi}_r(i,t)^2}{(1+\lambda^* d_{ii})^2} = \hat{\beta} \tag{13}$$

Where d_{ii} are diagonal elements of matrix $\mathbf{D}$ and $\boldsymbol{\varphi}_r = \mathbf{U}\widehat{\boldsymbol{\phi}}\mathbf{A}^T(\mathbf{A}\mathbf{A}^T)^{-1}\mathbf{A}$. $\mathbf{D}$ and $\mathbf{U}$ are singular value decompositions of the symmetric matrix $\mathbf{K}\mathbf{K}^T$such that $\mathbf{K}\mathbf{K}^T = \mathbf{U}^T\mathbf{D}\mathbf{U}$.

Solving problem **S1** is a bit trickier as there is a L_1-norm term in the goal function which is non-differential; however these problems can be solved easily (as demonstrated in the convex optimization problem literature [19]) using the soft threshold operator which is defined as follows:

$$\mathcal{T}_\eta(x) = \begin{cases} x - \eta & if \quad x \geq \eta \\ 0 & if \quad -\eta \leq x \leq \eta \\ x + \eta & if \quad x \leq -\eta \end{cases} \tag{14}$$

Where η is a positive number. The solution to an unconstrained minimization problem like problem **S2** is the following (performing simple partitioning and then differentiating the goal function) [19]:

$$\mathbf{Y}^{K+1} = \underset{\mathbf{Y}}{argmin} \| \mathbf{W}_d \odot \mathbf{Y} \|_1 + \frac{\lambda}{2}\|\mathbf{Y} - \mathbf{V}\mathbf{j}^{K+1}\|_{\mathcal{F}}^2 + \mathbf{u}^T(\mathbf{Y}^K - \mathbf{V}\mathbf{j}) = \mathcal{T}_{\frac{w_d}{\lambda}}(\mathbf{V}\mathbf{j}^{K+1} - \mathbf{u}^K) \tag{15}$$

Where w_d denotes the corresponding element of $\mathbf{W}_d$ and $\mathbf{Y}$. Solving problem **S1** might seem more difficult compared to **S2**, as we have a constraint to satisfy, but based on simple principles from optimization theory we know [19] that we can assume the constraint is not present and solve the problem and subsequently project the solution to the hyper-ellipsoid to satisfy the constraint (solving problem **P** for the obtained solution). Again, following simple partitioning, differentiating and projecting the solution to the hyper-ellipsoid, problem **S1** is solved as follows:

$$\mathbf{j}^{K+1} = \underset{\mathbf{j}}{argmin}\, \alpha \| \mathbf{W} \odot \mathbf{j} \|_1 + \frac{\lambda}{2}\|\mathbf{Y}^K - \mathbf{V}\mathbf{j}\|_{\mathcal{F}}^2 = \mathcal{P}_{\hat{\beta}}\left(\mathcal{T}_{\frac{\alpha.w}{\lambda.\mathcal{L}_v}}\left(\mathbf{j}^K - \frac{1}{\mathcal{L}_v}(\mathbf{V}^T\mathbf{V}\mathbf{j}^K - \mathbf{V}^T(\mathbf{Y}^K + \mathbf{u}^K))\right)\right) \tag{16}$$

Where $\mathcal{w}$ corresponds to the corresponding elements of **W** in the **j**, and $\mathcal{L}_{\mathcal{v}}$ is the Lipschitz constant of the function $f(x) = \mathbf{V}^T\mathbf{V}x$ (or basically the largest eigenvalue of $\mathbf{V}^T\mathbf{V}$). Thus, all the sub-problems, **S1**, **S2** and **P** can be solved efficiently and easily. The FISTA algorithm has a smart method for the updating rule of the variables that need to be updated at each iteration K (this is the internal iterations of the problem and not to be confused with outer iterations where the weights are updated). Putting together the solutions obtained for problem **S1** (Eq. 16), problem **S2** (Eq. 15) and the projection problem **P** (Eq.12 - Eq.13) we can propose the following algorithm to solve the optimization problem proposed in Eq. 5, i.e. fast spatio-temporal IRES:

1. Initialize with $\mathcal{t}_1 = 1, \tilde{\mathbf{j}}^1 = \mathbf{j}^0 = 0$ (or any desired initialization), $\mathbf{Y}^1 = \mathbf{V}\tilde{\mathbf{j}}^1 = 0$.
2. $\mathbf{j}^K = \mathcal{P}_{\widehat{\beta}}\left(\mathcal{T}_{\frac{\alpha.\mathcal{w}}{\lambda.\mathcal{L}_{\mathcal{v}}}}\left(\tilde{\mathbf{j}}^K - \frac{1}{\mathcal{L}_{\mathcal{v}}}(\mathbf{V}^T\mathbf{V}\tilde{\mathbf{j}}^K - \mathbf{V}^T(\mathbf{Y}^K + \mathbf{u}^K))\right)\right)$

   $$\mathcal{t}_{K+1} = \frac{1 + \sqrt{1 + 4\mathcal{t}_K{}^2}}{2}$$

   $$\tilde{\mathbf{j}}^{K+1} = \tilde{\mathbf{j}}^K + \left(\frac{\mathcal{t}_K - 1}{\mathcal{t}_{K+1}}\right).(\mathbf{j}^K - \mathbf{j}^{K-1})$$
3. $\mathbf{Y}^K = \mathcal{T}_{\frac{\mathcal{w}_d}{\lambda}}(\mathcal{V}\tilde{\mathbf{j}}^{K+1} - \mathbf{u}^K)$
4. $\mathbf{u}^{K+1} = \mathbf{u}^K + (\mathbf{Y}^{K+1} - \mathbf{V}\mathbf{j}^{K+1})$
5. Continue steps 2 to 4 at every iteration until we converge to a solution where **j** stabilizes and converges.

These simple steps outlined above will solve the spatio-temporal IRES formulated in Eq. 5. As it can be seen, all steps are very easy and efficient to perform and thus the algorithm is extremely efficient and fast. The introduction and use of the update rule parameter, $\mathcal{t}$, and the intermediate variable $\tilde{\mathbf{j}}$ at each iteration, is based on the FISTA algorithm. It is proven in [19] that this algorithm will be faster than simply updating the variables at each step without considering the values of **j** at previous iterations; thus converges to the optimal solution much faster than conventional methods and algorithms. The proposed FAST-IRES

is very fast and can be solved in a matter of seconds for problems we normally come across in EEG/MEG source imaging; that is usually data intervals of lengths which are in the order of seconds (T ~ 3,000 samples or more) and realistic problem sizes with couple of hundreds of electrodes/sensors (E ~ 100-200 measurements) and tens of thousands of unknowns (N ~ 15,000-25,000 variables or length of $\mathbf{j}$) for numerous temporal components (N_c ~ 10).

The FAST-IRES algorithm can be used for any source imaging problem to monitor the underlying and highly dynamic brain activities using non-invasive surface measurements. This can be extremely useful in identifying the location, extent and dynamics of seizures in patients with epilepsy to help guide for surgical resection of the epileptogenic zone or electrical grid placement, in medically intractable epilepsy patients or for studying epilepsy networks non-invasively.

Supplementary References.

1. Edelman, B. J., Baxter, B. & He, B. EEG source imaging enhances the decoding of complex right-hand motor imagery tasks. *IEEE Transactions on Biomedical Engineering* **63**, 4–14 (2016).
2. Bolstad, A., Van Veen, B. D. & Nowak, R. Space–time event sparse penalization for magneto-/electroencephalography. *NeuroImage* **46**, 1066–1081 (2009).
3. Ou, W., Hämäläinen, M. S. & Golland, P. A distributed spatio-temporal EEG/MEG inverse solver. *NeuroImage* **44**, 932–946 (2009).
4. Sohrabpour, A., Lu, Y., Worrell, G. & He, B. Imaging brain source extent from EEG/MEG by means of an iteratively reweighted edge sparsity minimization (IRES) strategy. *NeuroImage* **142**, 27–42 (2016).
5. Ding, L., Worrell, G. A., Lagerlund, T. D. & He, B. Ictal source analysis: localization and imaging of causal interactions in humans. *Neuroimage* **34**, 575–586 (2007).
6. Lu, Y., Yang, L., Worrell, G. A. & He, B. Seizure source imaging by means of FINE spatio-temporal dipole localization and directed transfer function in partial epilepsy patients. *Clinical Neurophysiology* **123**, 1275–1283 (2012).
7. Sohrabpour, A., Ye, S., Worrell, G. A., Zhang, W. & He, B. Noninvasive electromagnetic source imaging and granger causality analysis: An electrophysiological Connectome (eConnectome) approach. *IEEE Transactions on Biomedical Engineering* **63**, 2474–2487 (2016).
8. Kamiński, M. J., Ding, M., Truccolo, W. A. & Bressler, S. L. Evaluating causal relations in neural systems: Granger causality, directed transfer function and statistical assessment of significance. *Biological cybernetics* **85**, 145–157 (2001).
9. Akaike, H. A new look at the statistical model identification. *Automatic Control, IEEE Transactions on* **19**, 716–723 (1974).
10. Palus, M. & Hoyer, D. Detecting nonlinearity and phase synchronization with surrogate data. *Engineering in Medicine and Biology Magazine, IEEE* **17**, 40–45 (1998).
11. Theiler, J., Eubank, S., Longtin, A., Galdrikian, B. & Farmer, J. D. Testing for nonlinearity in time series: the method of surrogate data. *Physica D: Nonlinear Phenomena* **58**, 77–94 (1992).
12. Wilke, C., Ding, L. & He, B. Estimation of time-varying connectivity patterns through the use of an adaptive directed transfer function. *Biomedical Engineering, IEEE Transactions on* **55**, 2557–2564 (2008).
13. He, B. *et al.* eConnectome: A MATLAB toolbox for mapping and imaging of brain functional connectivity. *Journal of neuroscience methods* **195**, 261–269 (2011).
14. Babiloni, F. *et al.* Estimation of the cortical functional connectivity with the multimodal integration of high-resolution EEG and fMRI data by directed transfer function. *Neuroimage* **24**, 118–131 (2005).
15. Aydin, Ü. *et al.* Combined EEG/MEG Can Outperform Single Modality EEG or MEG Source Reconstruction in Presurgical Epilepsy Diagnosis. *PLOS ONE* **10**, e0118753 (2015).
16. Bast, T. *et al.* Noninvasive source localization of interictal EEG spikes: effects of signal-to-noise ratio and averaging. *J Clin Neurophysiol* **23**, 487–497 (2006).
17. Stefan, H. *et al.* Magnetic brain source imaging of focal epileptic activity: a synopsis of 455 cases. *Brain* **126**, 2396–2405 (2003).
18. Wieser, H. G. *et al.* Proposal for a New Classification of Outcome with Respect to Epileptic Seizures Following Epilepsy Surgery. *Epilepsia* **42**, 282–286 (2001).
19. Beck, A. & Teboulle, M. A Fast Iterative Shrinkage-Thresholding Algorithm for Linear Inverse Problems. *SIAM J. Imaging Sci.* **2**, 183–202 (2009).
20. Beck, A. & Teboulle, M. Fast Gradient-Based Algorithms for Constrained Total Variation Image Denoising and Deblurring Problems. *IEEE Transactions on Image Processing* **18**, 2419–2434 (2009).
21. Boyd, S., Parikh, N., Chu, E., Peleato, B. & Eckstein, J. Distributed optimization and statistical learning via the alternating direction method of multipliers. *Foundations and Trends® in Machine Learning* **3**, 1–122 (2011).
