# Supplementary material for: Noninvasive electromagnetic source imaging of spatiotemporally distributed epileptogenic brain sources
Source: Nat Commun. 2020 Apr 23;11:1946. doi: 10.1038/s41467-020-15781-0 (PMC7181775; doi:10.1038/s41467-020-15781-0)
Supplement: Supplementary file 3 — Reporting Summary [file 41467_2020_15781_MOESM3_ESM.pdf]

nature research

Corresponding author(s): Bin He

Last updated by author(s): Mar 19, 2020

Reporting Summary

Nature Research wishes to improve the reproducibility of the work that we publish. This form provides structure for consistency and transparency in reporting. For further information on Nature Research policies, seeAuthors & Referees and theEditorial Policy Checklist .

Statistics

For all statistical analyses, confirm that the following items are present in the figure legend, table legend, main text, or Methods section.

n/a	Confirmed	
☐	☒	The exact sample size (*n*) for each experimental group/condition, given as a discrete number and unit of measurement
☒	☐	A statement on whether measurements were taken from distinct samples or whether the same sample was measured repeatedly
☐	☒	The statistical test(s) used AND whether they are one- or two-sided<br>*Only common tests should be described solely by name; describe more complex techniques in the Methods section.*
☐	☒	A description of all covariates tested
☒	☐	A description of any assumptions or corrections, such as tests of normality and adjustment for multiple comparisons
☐	☒	A full description of the statistical parameters including central tendency (e.g. means) or other basic estimates (e.g. regression coefficient) AND variation (e.g. standard deviation) or associated estimates of uncertainty (e.g. confidence intervals)
☐	☒	For null hypothesis testing, the test statistic (e.g. *F*, *t*, *r*) with confidence intervals, effect sizes, degrees of freedom and *P* value noted<br>*Give P values as exact values whenever suitable.*
☒	☐	For Bayesian analysis, information on the choice of priors and Markov chain Monte Carlo settings
☒	☐	For hierarchical and complex designs, identification of the appropriate level for tests and full reporting of outcomes
☒	☐	Estimates of effect sizes (e.g. Cohen's *d*, Pearson's *r*), indicating how they were calculated

Our web collection on statistics for biologists contains articles on many of the points above.

Software and code

Policy information about availability of computer code

Data collection	The patient data was recorded in XLTeK amplifier in Mayo Clinic which is a typical software in clinical data collection. We used Curry 7/8 to view, select and filter the data and all other data analysis was done in MATLAB (2013b and 2018b). Details are presented in Methods and Code Availability sections.
Data analysis	Custom Software developed in MATLAB; Code is shared (https://github.com/bfinl/FAST-IRES) - All other toolboxes used are mentioned in the paper (EEGLab version 14.1.1b, specifically the infomax algorithm in this toolbox for independent component analysis, eConnectome version 1.0 beta for connectivity analysis, and custom code developed in MATLAB 2013b and also tested in 2018b, particularly a mathematical algorithm called FISTA was implemented by us which is available in the shared code - more details in Supplementary Methods). Detailed explanations are given in the manuscript under Code Availability.

For manuscripts utilizing custom algorithms or software that are central to the research but not yet described in published literature, software must be made available to editors/reviewers. We strongly encourage code deposition in a community repository (e.g. GitHub). See the Nature Research guidelines for submitting code & software for further information.

Data

Policy information about availability of data

All manuscripts must include a data availability statement. This statement should provide the following information, where applicable:

- Accession codes, unique identifiers, or web links for publicly available datasets
- A list of figures that have associated raw data
- A description of any restrictions on data availability

The data that support the findings of this study are available upon reasonable request from the corresponding author (B.H.). A partial subset of data has been de-identified and is available at (https://doi.org/10.35092/yhjc.11996931) for the benefit of the scientific community. Other data are not publicly available due to them containing information that could compromise research participant privacy/consent.

Field-specific reporting

Please select the one below that is the best fit for your research. If you are not sure, read the appropriate sections before making your selection.

☒ Life sciences ☐ Behavioural & social sciences ☐ Ecological, evolutionary & environmental sciences

For a reference copy of the document with all sections, see nature.com/documents/nr-reporting-summary-flat.pdf

Life sciences study design

All studies must disclose on these points even when the disclosure is negative.

Sample size	Detailed information is provided in the Methods section of the paper. To achieve localization errors that were comparable to ECoG grid size, i.e. distictively smaller than10mm, given our expectation and experience of localization errors of 5 mm for our approach, we determined a sample size of 25 to be adequate to guarantee a statistical power of 0.9 (alpha=0.05) in distinguishing the means of these distributions. Based on our experience and general trend on the field, analyzing the data of 20-25 patients gives us robust and reasonable results. We used all the patients that satisfied the inclusion criteria (described in Methods). We had 36 patients' data for analysis.
Data exclusions	Patients inclusion criteria are explained in Methods section. No data point was discarded.
Replication	Many patients (36 patients) were analyzed. Each patient has many tens of spikes and multiple seizures. The analysis was performed on these many instances and worked consistently.
Randomization	No randomization were used. The post-hoc statistics analysis was based on patients' seizure outcome.
Blinding	Blinding was not performed, because data included de-identified clinical reports of the patients, which was used for validation. These information were not used for analysis so blinding is irrelevant in this study.

Reporting for specific materials, systems and methods

We require information from authors about some types of materials, experimental systems and methods used in many studies. Here, indicate whether each material, system or method listed is relevant to your study. If you are not sure if a list item applies to your research, read the appropriate section before selecting a response.

Materials & experimental systems

n/a	Involved in the study	
☒	☐	Antibodies
☒	☐	Eukaryotic cell lines
☒	☐	Palaeontology
☒	☐	Animals and other organisms
☐	☒	Human research participants
☐	☒	Clinical data

Methods

n/a	Involved in the study	
☒	☐	ChIP-seq
☒	☐	Flow cytometry
☒	☐	MRI-based neuroimaging

Human research participants

Policy information about studies involving human research participants

Population characteristics	Detailed information about inclusion criteria is brought in the Methods section of the paper, additionally, patients' (de-identified) information are brought in details in Supplementary Tables 4 and 5.
Recruitment	The patients were referred to Mayo Clinic, Rochester for treatment. Focal epilepsy patients who underwent intra-cranial recordings and/or had surgery were included in the study (details in the paper). This procedure is part of the medical routine performed at Mayo Clinic by Dr. Worrell's team. No known bias is identified in treating and collecting data from these patients.
Ethics oversight	The patients participated in the study willingly and gave written consent. The institutional review boards of Mayo Clinic, Carnegie Mellon University and University of Minnesota approved the study and all research was conducted within the regulations set by these IRBs.

Note that full information on the approval of the study protocol must also be provided in the manuscript.

Clinical data

Policy information about clinical studies

All manuscripts should comply with the ICMJE guidelines for publication of clinical research and a completed CONSORT checklist must be included with all submissions.

Clinical trial registration	This is not a clinical trial but ongoing routine clinical treatment at Mayo Clinic.
Study protocol	This is not a clinical trial but rather patients undergoing treatment routine.
Data collection	Data were collected at a hospital, Mayo Clinic, as part of a routine medical treatment for medically intractable focal epilepsy patients candidate for surgery (to treat seizures).
Outcomes	The outcome is defined as seizure-freedom rate based on a widely recognized rating system, namely the international league against epilepsy (ILAE). This scoring system measures the reduction rate of seizures and seizure-freedom rates in patients who underwent treatment, i.e. surgery. Ranges from totally seizure-free patients, i.e. ILAE 1, to various conditions of seizure rates in these patients (full details and definitions in supplementary - specifically Supplementary Tables 4 and 5).
