# Supplementary material for: Noninvasive electromagnetic source imaging of spatiotemporally distributed epileptogenic brain sources
Source: Nat Commun. 2020 Apr 23;11:1946. doi: 10.1038/s41467-020-15781-0 (PMC7181775; doi:10.1038/s41467-020-15781-0)
Supplement: Supplementary file 4 — Description of Additional Supplementary Files [file 41467_2020_15781_MOESM4_ESM.pdf]

Description of Additional Supplementary Files

File Name: Supplementary Movie 1
Description: **Simulation Case Study.** In this video a simulated case-study, with three sources activated simultaneously, are depicted (top) with the corresponding estimated solution (bottom) for an interval of 1.5 seconds. Time-courses of activity for estimated and simulated sources are shown at the right panel. The signal-to-noise ratio in this simulated example is 10dB (the same example discussed in Supplementary Fig. 3 and Supplementary Table 3).

File Name: Supplementary Movie 2
Description: **Spike Imaging Example**. Spike imaging results on a left temporal lobe epilepsy patient is shown in this video. In a 0.5 second interval around spike peak-time, the solution (spatiotemporal distribution of the underlying sources) is shown in a left and right view. The mean global field power (MGFP) of the EEG channels is shown in the bottom panel as a reference. The electrocorticogram (ECoG) electrodes implanted in this patient are depicted as black circles and the red circles are ECoG electrodes denoted as seizure onset zone by epileptologists.

File Name: Supplementary Movie 3
Description: **Ictal Imaging Example (I).** Ictal imaging results in a left temporal lobe epilepsy patient is presented from a left view and a right view during a three second interval at seizure onset. The mean global field power (MGFP) of the EEG channels is shown in the bottom panel as a reference. The right view (right panel) is a mesial view of the temporal lobe. Most of the right lobe and considerable parts of the left hemisphere have been removed so that a better inspection of the deep mesial structure of the left temporal lobe is possible. The ECoG/depth electrodes implanted in this patient are depicted as black circles and the red circles are ECoG/depth electrodes denoted as seizure onset zone by epileptologists. The intra-cranial EEG (iEEG) traces (in blue) and electrophysiological source imaging (ESI) time-courses of activity from FASTIRES (in red) are also depicted on the right panel. Applying this computational approach enables us to noninvasively inspect electrical activity in deep tissue. Note the red electrodes, which are deep intracranial EEG electrodes, indicated as seizure onset electrodes. Electrodes in the left anterior temporal (LAT) region and the mesial hippocampal (HC) regions were denoted as seizure onset zone by epileptologists. The estimated time-course of activity from FAST-IRES is compared to iEEG traces in the LAT and HC regions in this video.

File Name: Supplementary Movie 4
Description: **Ictal Imaging Example (II).** Ictal imaging results in a left fronto-parietal lobe epilepsy patient is presented from a left view and a right view during a three second interval at seizure onset. The mean global field power (MGFP) of the EEG channels is shown in the bottom panel as a reference. The ECoG electrodes implanted in this patient are depicted as black circles and the red circles are ECoG electrodes denoted as seizure onset zone by epileptologists. Magenta circles denote electrodes that seizure activity spread to, quickly after onset, as reported by epileptologists. Note how the seizure activity can propagate to the ipsilateral occipital and frontal lobe as well as the contralateral temporal lobe. This emphasizes the importance of applying directional connectivity measures to determine the network dynamics and structure, more accurately and quantitively.
